# Evidence that TSC2 acts as a transcription factor and binds to and represses the promoter of Epiregulin

**DOI:** 10.1093/nar/gku278

**Published:** 2014-04-19

**Authors:** Shalmali Avinash Pradhan, Mohammad Iqbal Rather, Ankana Tiwari, Vishwanath Kumble Bhat, Arun Kumar

**Affiliations:** Department of Molecular Reproduction, Development and Genetics, Indian Institute of Science, Bangalore 560012, India

## Abstract

The *TSC2* gene, mutated in patients with tuberous sclerosis complex (TSC), encodes a 200 kDa protein TSC2 (tuberin). The importance of TSC2 in the regulation of cell growth and proliferation is irrefutable. TSC2 in complex with TSC1 negatively regulates the mTOR complex 1 (mTORC1) via RHEB in the PI3K-AKT-mTOR pathway and in turn regulates cell proliferation. It shows nuclear as well as cytoplasmic localization. However, its nuclear function remains elusive. In order to identify the nuclear function of TSC2, a whole-genome expression profiling of TSC2 overexpressing cells was performed, and the results showed differential regulation of 266 genes. Interestingly, transcription was found to be the most populated functional category. *EREG* (Epiregulin), a member of the epidermal growth factor family, was found to be the most downregulated gene in the microarray analysis. Previous reports have documented elevated levels of *EREG* in TSC lesions, making its regulatory aspects intriguing. Using the luciferase reporter, ChIP and EMSA techniques, we show that TSC2 binds to the *EREG* promoter between −352 bp and −303 bp and negatively regulates its expression. This is the first evidence for the role of TSC2 as a transcription factor and of TSC2 binding to the promoter of any gene.

## INTRODUCTION

Mutations in the *TSC2* (tuberous sclerosis 2) gene cause an autosomal dominant disorder, tuberous sclerosis complex (TSC) (1). TSC2 codes for a 200 kDa protein of 1807 amino acids (1,2), which contains a leucine zipper region (amino acids 75–107), two coiled-coil domains (amino acids 346–371 and 1008–1021), two transcriptional activation domains (amino acids 1163–1259 and 1690–1743), a conserved GTPase activating protein homology region (amino acids 1517–1674) and a nuclear localization signal (NLS; amino acids 1743–1755) (3). It localizes to both cytoplasm as well as the nucleus (4). It interacts with TSC1 (tuberous sclerosis 1) and forms the TSC1/TSC2 (hamartin/tuberin) protein complex (2). Mutations in the *TSC1* gene also cause TSC (5). *TSC1* codes for a 1164 amino acids long cytoplasmic protein of 130 kDa (2,3). The TSC1/TSC2 complex is a key negative regulator of the PI3K-AKT-mTOR pathway. It has a dual role and acts by either inhibiting mTORC1 (mTOR complex 1) or by positively regulating mTORC2 (mTOR complex 2) (6,7). mTORC1 and mTORC2 are functionally distinct complexes and are important for nutrient and growth factor signaling. mTORC1 phosphorylates EIF4EBPs (eukaryotic translation initiation factor 4E binding proteins) and RPS6KB1 (ribosomal protein S6 kinase, 70kDa, polypeptide 1; S6K1), whereas mTORC2 phosphorylates SGK1 (serum/glucocorticoid regulated kinase 1) and AKT (v-akt murine thymoma viral oncogene) (6–8). Although TSC1/TSC2 complex negatively regulates mTORC1, overexpression of TSC1/TSC2 has been shown to activate AKT via mTORC2 and in turn the downstream players of the PI3K-AKT-mTOR pathway like S6K1 (ribosomal protein S6 kinase 1) (7). Thus, the balance between inhibition of mTORC1 and activation of mTORC2 by the TSC1/TSC2 complex regulates the PI3K-AKT-mTOR pathway.

Physiological roles of TSC1 and TSC2, which are independent of the PI3K-AKT-mTOR pathway, are termed as ‘non-canonical’ (9). Transcription is one such independent function of TSC2 (9), which explains its nuclear localization, but remains poorly understood (4). The speculation that TSC2 has transcription-related functions is also based on the identification of transcriptional activation domains in its C-terminal region. Further, TSC2 directly interacts with and modulates transcription mediated by members of the steroid receptor superfamily (10). It was also shown that TSC2 binds specifically to ERα (estrogen receptor alpha) and inhibits estrogen induced proliferation by opposing the activation of PDGFR-β (platelet-derived growth factor receptor beta) and ERK1/2 (extracellular-signal-regulated kinase 1/2) (11). Thus, the above studies have shown that TSC2 modulates transcription, but it has not been shown yet to act as a transcription factor and bind to DNA.

In order to identify the nuclear function of TSC2, we used a genome-wide expression microarray of TSC2 overexpressing cells and identified *EREG* (Epiregulin), a member of the epidermal growth factor (EGF) family, as the most downregulated gene. Using the luciferase reporter, chromatin-immunoprecipitation (ChIP) and electrophoretic mobility shift assay (EMSA) techniques, we provide the first evidence that TSC2 also functions as a transcription factor and represses the expression of *EREG* directly via binding to its promoter.

## MATERIALS AND METHODS

### Cell culture and generation of stable clones

The KB (human oral squamous cell carcinoma) cell line was procured from the National Cell Repository at the National Centre for Cell Sciences, Pune, India. The SCC131 (human oral squamous cell carcinoma) cell line was a kind gift from Prof. Susanne M. Gollin, University of Pittsburgh, Pittsburgh, PA, USA. Cells were grown in Dulbecco's Modified Eagle's Medium (DMEM) supplemented with 10% fetal bovine serum and 1× antibiotic-antimycotic solution (all from Sigma-Aldrich, St Louis, MO, USA) at 37°C in 5% CO_2_.

The empty vector pcDNA3.1(+) and the pcDNA3.1(+)/TSC2 construct harboring a full-length human *TSC2* gene (a kind gift from Dr Elizabeth P. Henske, Brigham and Women's Hospital, Boston, MA, USA) were transfected separately in KB cells, using the Effectene^®^ transfection reagent (Qiagen, Hilden, Germany) according to the manufacturer's protocol. Selection of transfected cells was started 48 h post transfection with 1 mg/ml of G418 (Calbiochem^®^, Darmstadt, Germany) and continued until colonies were visible and well isolated from each other. Individual colonies were picked with cloning cylinders and cultured as independent stable clones. The stable TSC2 overexpressing clones or a vector control clone were selected using western blotting and validated by semi-quantitative real time-polymerase chain reaction (RT-PCR) as described below.

### Western blot analysis

Total protein lysates were prepared using the CellLytic^TM^ M Cell Lysis Reagent supplemented with 1X Protease Inhibitor Cocktail (all from Sigma-Aldrich, St Louis, MO, USA). Equal amounts of proteins were resolved on a sodium dodecyl sulphate-polyacrylamide gel electrophoresis (SDS-PAGE) and then transferred onto a Polyvinylidene fluoride (PVDF) membrane (Millipore, Darmstadt, Germany). Anti-TSC2 (cat# sc-893, Santa Cruz Biotechnology, Dallas, TX, USA), anti-TSC1 (cat# sc-12082, Santa Cruz Biotechnology, Dallas, TX, USA), anti-SGK1 (cat# ab59337, Abcam plc, Cambridge, UK), anti-SGK1 (phosphor S422) (cat# ab55281, Abcam plc, Cambridge, UK), anti-EREG (cat# ab175118, Abcam plc, Cambridge, UK), anti-p-pS6K1 (Thr389) (cat# SC-11759, Santa Cruz Biotechnology, Dallas, TX, USA), anti-pS6K1 (cat# sc-230, Santa Cruz Biotechnology, Dallas, TX, USA) and β-actin (product# A-5441, Sigma-Aldrich, St Louis, MO, USA) antibodies were used for the western blot analysis. Signals on western blots were detected using HRP-conjugated secondary antibodies (Bangalore Genei, Bangalore, India), Immobilon Western Chemiluminescent HRP (Horseradish peroxidase) Substrate (Millipore, Darmstadt, Germany) and X-ray films (Kodak, Rochester, NY, USA). β-actin was used as a loading control in western blotting.

### Total RNA isolation

Total RNA was isolated from cells using the RNeasy Mini Kit (Qiagen, Hilden, Germany) according to the manufacturer's protocol. Total RNA was quantitated using the NanoDrop 1000 spectrophotometer (Thermo Scientific, Wilmington, DE, USA).

### Immunofluorescence staining

Cells from stable clones were grown overnight on 22 mm^2^ cover slips, and the subcellular localization of TSC2 was investigated using a rabbit polyclonal anti-TSC2 antibody (product# T9574, Sigma-Aldrich, St Louis, MO, USA) at 1:25 dilution. Staining was visualized using a rabbit-cy5 (Fermentas, Burlington, Canada) secondary antibody. Cells were counterstained with 1 μg/ml DAPI (Sigma-Aldrich, St Louis, MO, USA), and the images were captured using a confocal Zeiss LSM 510 META microscope at 60X magnification.

### Microarray protocols and data analysis

Microarray experiments were performed using a whole human genome (4 × 44K) oligonucleotide expression array (Agilent Technologies, Santa Clara, CA, USA). Two clones, P and G, overexpressing TSC2 constituted the biological duplicates and each clone was in turn processed in duplicate, representing technical duplicates. Total RNA (500 ng) samples from each of the two TSC2 overexpressing clones and a vector control clone V were labeled separately using the Low RNA Input Linear Amplification Kit (Agilent Technologies, Santa Clara, CA, USA) according to the manufacturer's protocol. Samples with specific activity of >8 pmol Cy3 or Cy5/mg cRNA were selected for competitive hybridization. The Cy5 labeled cRNA (825 ng) from each of the two TSC2 overexpressing clones was mixed separately with the Cy3 labeled cRNA (825 ng) from the vector control clone. Each of these mixes was allowed to hybridize with a single array on the 4 × 44K slide. Altogether four such hybridizations were performed, two for each of the two TSC2 overexpressing clones. Hybridization was carried out in a hybridization oven on a rotisserie (four rotations per minute) at 65°C for 17 h. After hybridization, the microarray slide was washed using the gene expression wash buffer kit (Agilent Technologies, Santa Clara, CA, USA), dried at room temperature and scanned using the Agilent Scanner G2505C. The microarray image analysis was performed using the feature extraction version 10.5.1.1 software (Agilent Technologies, Santa Clara, CA, USA). The background corrected raw intensity values were used for analysis. Normalization was performed using the LOWESS algorithm (http://www.improvedoutcomes.com/docs/WebSiteDocs/PreProcessing/Normalization/Two_Color_Datasets/Overview_of_Lowess_Normalization.htm). Differential expression of genes was calculated by averaging the normalized technical duplicates and comparing them with the biological duplicate. The MeV version 4.6 software (Dana-Farber Cancer Institute, Boston, MA, USA) was used to sort statistically significant differentially expressed genes. Statistical filters were applied by using a cutoff value of *P* < 0.05 after performing a paired t-test on the technical replicates. Fold changes were calculated as follows: fold change = Cy5 mean processed value - Cy3 mean processed value, where Cy5 or Cy3 mean processed value represents the mean of the log-transformed (base-2) probe set values of the technical replicates. Fold change of ≥1.5 was taken as a cut off value (12). The fold change was expressed as an average of normalized log-transformed values for technical duplicates. The microarray data have been deposited in the Gene Expression Omnibus (GEO) repository (http://www.ncbi.nlm.nih.gov/geo/) with accession number GSE52061.

To examine the molecular function and networks, the microarray data analysis was performed by the DAVID v6.7 software (Database for Annotation, Visualization and Integrated Discovery) (http://david.abcc.ncifcrf.gov/) (13,14) and the Ingenuity Pathways Analysis tool, IPA version 8.7 (Ingenuity H Systems Inc., Redwood City, CA, USA; http://www.ingenuity.com), which enabled the identification of biological mechanisms/functions and networks most relevant to differentially expressed genes.

### First-strand cDNA preparation, semi-quantitative and qRT-PCR analyses

The first-strand cDNA was synthesized from 2 μg of total RNA using the RevertAid^TM^ H Minus First Strand cDNA Synthesis Kit (Fermentas, Burlington, Canada) in a 20 μl reaction volume as per the manufacturer's protocol. For semi-quantitative and qRT-PCR, *RPL35A* was used as a normalizing control (15) to equalize the amount of cDNA generated from each sample. Following equalization of *RPL35A* RT-PCR products, the same amounts of cDNA templates were used in RT-PCR to check for the expression of *EREG* or *TSC2*. RT-PCR products were resolved by agaroge gel electrophoresis and visualized by ethidium bromide staining on an UV trans-illuminator (Bangalore Genei, Bangalore, India).

The Dynamo SYBR Green Mix (Finnzymes, Espoo, Finland) was used for qRT-PCR analysis in an ABIprism^®^ 7900HT sequence detection system (Applied Biosystems, Foster City, CA, USA). The analysis was performed using the SDS 2 software (Applied Biosystems, Foster City, CA, USA). The fold change was calculated using the following equation (16): ΔC_t(*TSC2* overexpressing clone)_ = C_t (gene)_ – C_t (*RPL35A*)_ and ΔC_t(Vector clone)_ = C_t(gene)_ – C_t (*RPL35A*)_. Where C_t_ is the cycle threshold value, and ΔC_t_ represents the gene expression normalized to *RPL35A*. The statistical significance of the difference in mRNA expression levels was assessed by two-tailed unpaired t-test with Welch's correction using the GraphPad PRISM5 software (GraphPad Software Inc., San Diego, CA, USA). A probability value of *P* ≤ 0.05 was considered to be significant. Details of different RT-PCR primers are given in Supplementary Table S1.

### RNA interference

The ON-TARGETplus siRNA sequences specifically targeting human *TSC1* and *TSC2* were purchased from Dharmacon Thermo Fisher Scientific (Lafayette, CO, USA). Transient transfection of siRNA was performed using Lipofectamine^TM^ 2000 (Life Technologies, Carlsbad, CA, USA), as recommended by the manufacturer. Control cells were treated with 100 nM scrambled siRNA (mock). After 72 h, cells were lysed for RNA and protein isolation to assess the gene expression and binding of TSC2 to the *EREG* promoter.

### Generation of *EREG* promoter and TSC2 NLS deletion constructs

The *EREG* promoter has been reported previously between −875 bp and +165 bp (17). In order to generate *EREG* promoter deletion constructs in the pGL3-Basic vector (Promega, Madison, WI, USA) for determining the effect of TSC2 on the *EREG* promoter activity, primers for pFP1EREG, pFP2EREG and pFP3EREG constructs were adapted from Ornskov *et al.* (17). Further, ∼1.5 kb upstream sequence from the transcription start site of *EREG* harboring its promoter (17) was retrieved from the UCSC Genome Bioinformatics site (http://genome.ucsc.edu). The sequence was used to design a primer set (Supplementary Table S2) to generate the pFP4EREG construct by PCR, using a standard laboratory procedure and total human genomic DNA as a template. Amplified fragments were first cloned in a TA cloning vector pTZ57R (Fermentas, Burlington, Canada) and after verifying the inserts by sequencing on an ABIprism A310 automated sequencer (PE Biosystems, Foster City, CA, USA) subcloned in the pGL3-Basic vector upstream to the luciferase reporter gene (Luc) to generate deletion constructs. Incorporation of different restriction enzyme sites in forward and reverse primers allowed directional cloning of the insert in the pGL3-basic vector (Supplementary Table S2). The pFP1EREG-mut construct harboring the 50 bp deletion was generated by site-directed mutagenesis as described in Rather *et al.* (18), using a pair of primers (Supplementary Table S2). The pcDNA3.1(+)/TSC2-NLSdel construct harboring the deleted NLS amino acid sequence (RLRHIKRLRQRIR) was also generated by site-directed mutagenesis as described in Rather *et al.* (18), using a pair of primers (Supplementary Table S2) and a full-length TSC2 construct as a template.

### Transfection and reporter gene assay

Cells from TSC2 overexpressing and vector control clones were seeded in 24-well plates at a density of 6 × 10^4^ cells/well a day prior to the transfection. Eight hundred nanogram of each of the promoter deletion constructs (pFP1EREG, pFP2EREG, pFP3EREG and pFP4EREG) was used for transfection. The pRL-TK vector (Promega, Madison, WI, USA), harboring the Renilla luciferase gene, was co-transfected as an internal control for normalization of transfection efficiency. The pGL3-Control vector harbors the SV40 promoter and enhancer sequences, resulting in a strong expression of the luciferase gene. Hence, this construct was used to monitor the transfection efficiency and acted as a positive control for the luciferase reporter assay.

For transient co-transfection of pcDNA3.1(+)/TSC2 and *EREG* promoter deletion constructs, KB or SCC131 cells were seeded at a density of 6 × 10^4^ cells/well in 24-well plates a day prior to the transfection. Five hundred nanogram of each of the promoter deletion constructs and 300 ng of either pcDNA3.1(+) or the pcDNA3.1(+)/TSC2 construct were used for co-transfection. The pRL-TK vector was also co-transfected as an internal control. The luciferase reporter gene activity was measured using the Dual Luciferase^®^ Reporter Assay System (Promega, Madison, WI, USA) and the VICTOR™ *X* Multilabel Plate Reader (PerkinElmer, Waltham, MA, USA). The statistical significance was assessed by two-tailed unpaired t-test with Welch's correction using the GraphPad PRISM5 software (GraphPad Software Inc., San Diego, CA, USA). A probability value of *P* < 0.05 was considered to be significant.

### ChIP

The ChIP analysis was performed as described previously by Bajaj *et al.* (19). Briefly, SCC131 cells were treated with formaldehyde to cross-link proteins and DNA. Cells were lysed in a cell lysis buffer (5 mM PIPES, 85 mM KCl, 0.5% NP40, 1X Protease Inhibitor Cocktail and 2 mM PMSF) and sonicated using a Branson Sonicator (Best Labs Deals Inc., Garner, NC, USA) to shear the DNA. The lysate was precleared using a mixture of protein G beads (GE Healthcare, Little Chalfont, UK), bovine serum albumin and salmon sperm DNA in order to remove non-specific binding to the beads. It was then divided into three aliquots. One aliquot was incubated with the antibody of interest (+Ab), i.e. an anti-rabbit TSC2 antibody (product# T9574, Sigma-Aldrich, St Louis, MO, USA). The second aliquot was incubated without an antibody (-Ab) and served as a negative control. The third aliquot was used as an input DNA (INP) and acted as a positive control in PCR. PCR was performed with primers specific to the *EREG* promoter (Supplementary Table S3). The promoter enrichment of *EREG* by ChIP assay was also quantified using RT-PCR to assess the specific enrichment of its promoter sub-regions. The RT-PCR was performed using EREGF/EREGR and EREGF1/EREGR1 primers (Supplementary Table S3). The fold enrichment was calculated using 2^−ΔΔCt^ method: ΔC_t_ = C_t_(ChIP DNA)-C_t_(input DNA) and ΔΔC_t_ = ΔC_t_ of sample (immunoprecipitated DNA of sub-region 1 or 2)−ΔC_t_ of IgG control [SABiosciences, Valencia CA, USA; http://www.sabiosciences.com/].

Since TSC2 has not been shown to bind to DNA and a positive control for TSC2-DNA interaction was not available, the DNA binding ability of the SP1 transcription factor was utilized as a surrogate positive control, and the PCR was performed with primers specific to the SP1 promoter of the *SLC22A18AS* gene (Supplementary Table S3) (19). For ChIP, the anti-SP1 antibody (product# S-9809) was purchased from Sigma-Aldrich (St Louis, MO, USA).

### EMSA

Probes for EMSA were generated by end-labeling of annealed duplex oligonucleotides using T4 polynucleotide kinase and γ-P^32^ATP (3000 ci/mmole; BRIT, Hyderabad, India). Probes were suitably diluted to obtain 25 000 cpm/μl. Probes used for EMSA have been listed in Supplementary Table S4. Nuclear extracts from SCC131 cells were prepared in a nuclei lysis buffer [10 mM Tris-HCl (pH 7.4), 4 mM EDTA, 30 mM KCl, 1% NP-40, 1 mM DTT, 100 μM sodium ortho-vanadate and 1X protease inhibitor cocktail] (all from Sigma-Aldrich, St Louis, MO, USA), and the protein was quantitated by the Bradford assay. DNA–protein binding reactions were carried out in a 25 μl volume using 4 μg of nuclear extract in a binding buffer containing 20 mM HEPES (pH 7.5), 60 mM KCl, 0.2 mM EDTA, 10% glycerol, 1 mM DTT and 1X Protease Inhibitor Cocktail (all from Sigma-Aldrich, St Louis, MO, USA). To analyze the supershift, 4 μg of an anti-TSC2 antibody (product# T9574, Sigma-Aldrich, St Louis, MO, USA) was added 15 min prior to the addition of an end-labeled probe. Subsequently, 50 000 cpm of an end-labeled probe was added and incubated for 20 min. Following incubation, DNA–protein complexes were resolved on 5% non-denaturing polyacrylamide gels in 0.5× TBE (Tris-borate EDTA) buffer at 4°C and 50V. Gels were vacuum dried and subjected to Typhoon phosphor imager analysis (GE Technologies, Little Chalfont, UK).

### Treatment of cells with rapamycin

The mTOR inhibitor, rapamycin (Sigma-Aldrich, St Louis, MO, USA) was dissolved in DMSO (Sigma-Aldrich, St Louis, MO, USA) and stored at −20°C. Cells from stable clones were seeded separately a day prior to the treatment in 25 cm^2^ flasks (6 × 10^6^ cells/flask). Cells were either treated with 100 nM rapamycin or with an equivalent volume of DMSO (vehicle control) for 24 h in the complete DMEM. Post treatments, the cells were harvested and either used to prepare total protein lysates for the western blot analysis or for the isolation of total RNA for determining the level of *EREG* as described above.

### Identification of a conserved binding motif in promoters of TSC2 regulated genes

In order to identify a conserved motif in the promoters of TSC2 regulated genes, we first retrieved the promoter sequences of 24/40 (top 20 upregulated and top 20 downregulated) genes from the Transcriptional Regulatory Element Database (TRED; http://rulai.cshl.edu/cgi-bin/TRED/tred.cgi?process=promInfo&pid=31498). Promoter sequences for the remaining 16/40 genes were not available in TRED. The promoter sequence of each gene was then aligned with the *EREG* probe 11 sequence by the ClustalW2 program (http://www.ebi.ac.uk/Tools/msa/clustalw2/) to find a conserved TSC2 binding motif.

## RESULTS

### Generation of TSC2 overexpressing stable clones and microarray analysis

**Figure 1.**
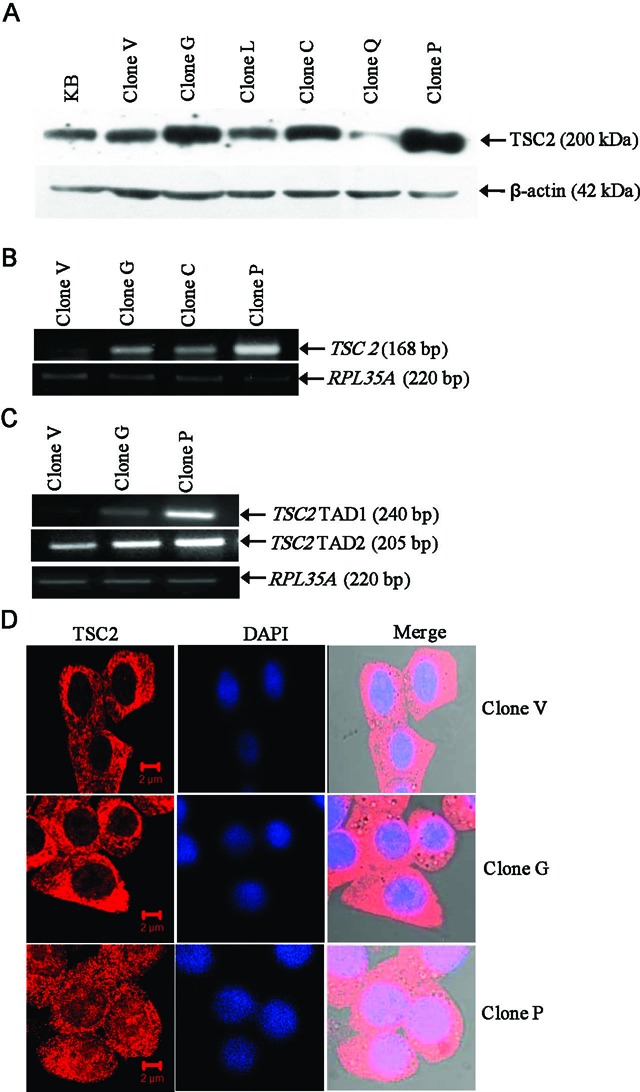
Identification of TSC2 overexpressing stable clones. (**A**) Screening of TSC2 overexpressing clones by western blot analysis. The stable clone V harbors the empty vector pcDNA3.1(+). Clones G, L, C, Q and P harbor a full-length TSC2 construct pcDNA3.1(+)/TSC2. KB represents parental cells used in the generation of stable clones. β-actin was used as a loading control. (**B**) Semi-quantitative RT-PCR analysis to further confirm the overexpression of *TSC2* in stable clones. *RPL35A* was used as a normalizing control. (**C**) Semi-quantitative RT-PCR analysis to determine the overexpression of *TSC2* TAD1 and TAD2. (**D**) Immunofluorescence analysis to assess the overexpression of TSC2 in stable clones G and P in comparison to clone V.

**Figure 2.**
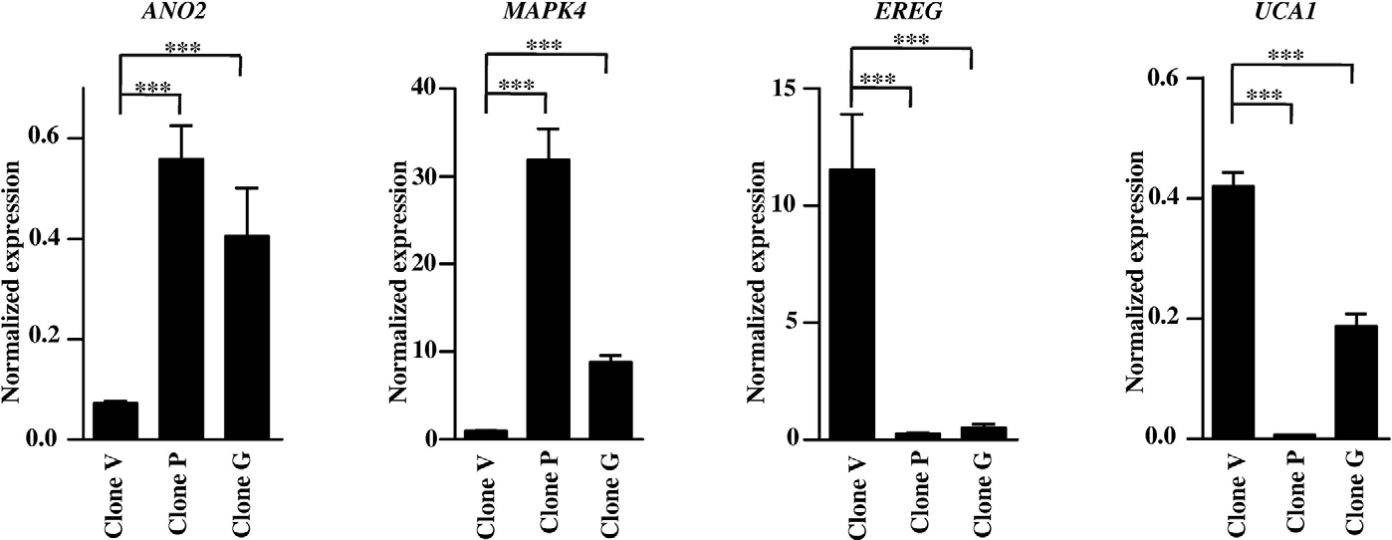
Validation of microarray data by qRT-PCR. As observed in microarray experiments, *ANO2* and *MAPK4* were significantly upregulated, and *EREG* and *UCA1* were significantly downregulated in TSC2 overexpressing clones P and G as compared to the vector clone V. Each bar represents the mean ± SD of triplicate experiments performed twice. ***P < 0.001.

Following transfection of the pcDNA3.1(+)/TSC2 construct in KB cells and G418 selection, we identified three TSC2 overexpressing clones (viz., G, C and P), using western blotting and RT-PCR (Figure 1A and B). We also transfected the pcDNA3.1(+) vector in KB cells and generated a stable vector clone V (Figure 1A), which was used in microarray experiment as a control and showed the basal level of TSC2 expression (compare KB cells with clone V). Two of the clones, P and G, were subsequently used in microarray experiments as they showed the highest level of TSC2 expression (Figure 1A and B). These two clones also showed an increased expression of TSC2 transcription activation domain (TAD) 1 and 2 in comparison to the vector clone V (Figure 1C). Further, the immunofluorescence analysis showed an increased expression of TSC2 in the cytoplasm as well as in the nucleus of cells from clones P and G in comparison to the vector clone V (Figure 1D). Microarray analysis using two biological replicates (clones P and G) and the vector clone V revealed significant upregulation of 106 and downregulation of 160 genes (Supplementary Table S5) in both the TSC2 overexpressing clones in comparison to the vector control clone V. Supplementary Tables S6 and S7 enlist the top 20 upregulated and top 20 downregulated genes, respectively. We randomly selected four genes (viz., *ANO2*, *MAPK4*, *EREG* and *UCA1*) and validated the microarray data by the qRT-PCR analysis. As expected, qRT-PCR results validated the microarray data and the magnitude of the effect on gene expression was consistent with the TSC2 expression level (Supplementary Tables S6 and S7, Figure 2).

### Functional annotation and network analysis of differentially expressed genes

**Figure 3.**
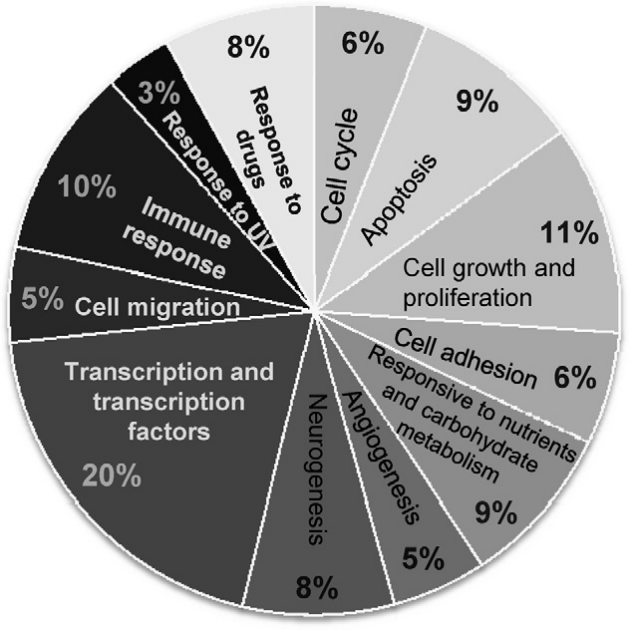
Functional annotation of the genes regulated by TSC2. Pie chart represents the percentage of genes annotated to each functional category. Functional categories were assigned by the DAVID software.

All the genes found to be significantly differentially regulated by TSC2 overexpression in both the biological replicates were analyzed by the DAVID and IPA software, which led to the identification of many functional categories (Figure 3, Supplementary Figure S1 and Supplementary Tables S8–S19). Interestingly, the most populated functional categories were the transcription associated functions (Figure 3, Supplementary Tables S8 and S9). Our interest in analyzing the genes regulated by TSC2 was directed at delineating its nuclear function (transcription), which drew our attention to the gene *EREG* (Epiregulin) as it was drastically downregulated in microarray experiments (−4.75-fold in clone P and −3.49 -fold in clone G) (Supplementary Table S7).

### Knockdown of TSC2 induces EREG expression

**Figure 4.**
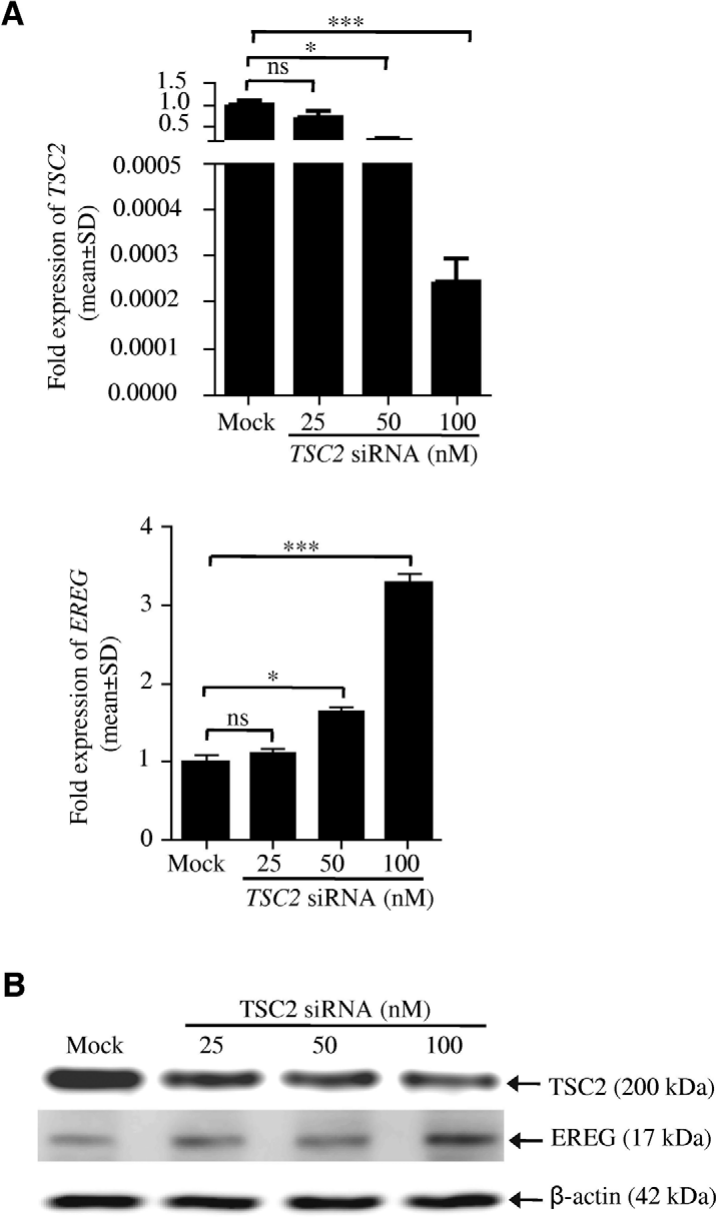
Knockdown of TSC2 induces EREG expression. (**A**) qRT-PCR analysis of *TSC2* and *EREG* in SCC131 cells transiently transected with TSC2 siRNA. Note, the knockdown of TSC2 in a dose-dependent manner (top panel) leads to a concomitant induction of the EREG expression (bottom panel). Mock represents the cells transfected with the scrambled siRNA. (**B**) Effect of TSC2 knockdown on EREG expression using western blotting. Note, knockdown of TSC2 protein in a dose-dependent manner (top panel) leads to a concomitant induction of EREG expression (middle panel). β-actin was used as a normalizing control (bottom panel). ns, data non-significant, *P < 0.05 and ***P < 0.001.

Further to underscore our finding that TSC2 negatively regulates EREG expression, the siRNA-mediated knockdown of TSC2 was performed in SCC131 cells and its effect on the EREG expression was analyzed by qRT-PCR and western blotting. As expected, TSC2 knockdown induced the expression of EREG in SCC131 cells (Figure 4).

### TSC2 regulates *EREG* at the transcriptional level

**Figure 5.**
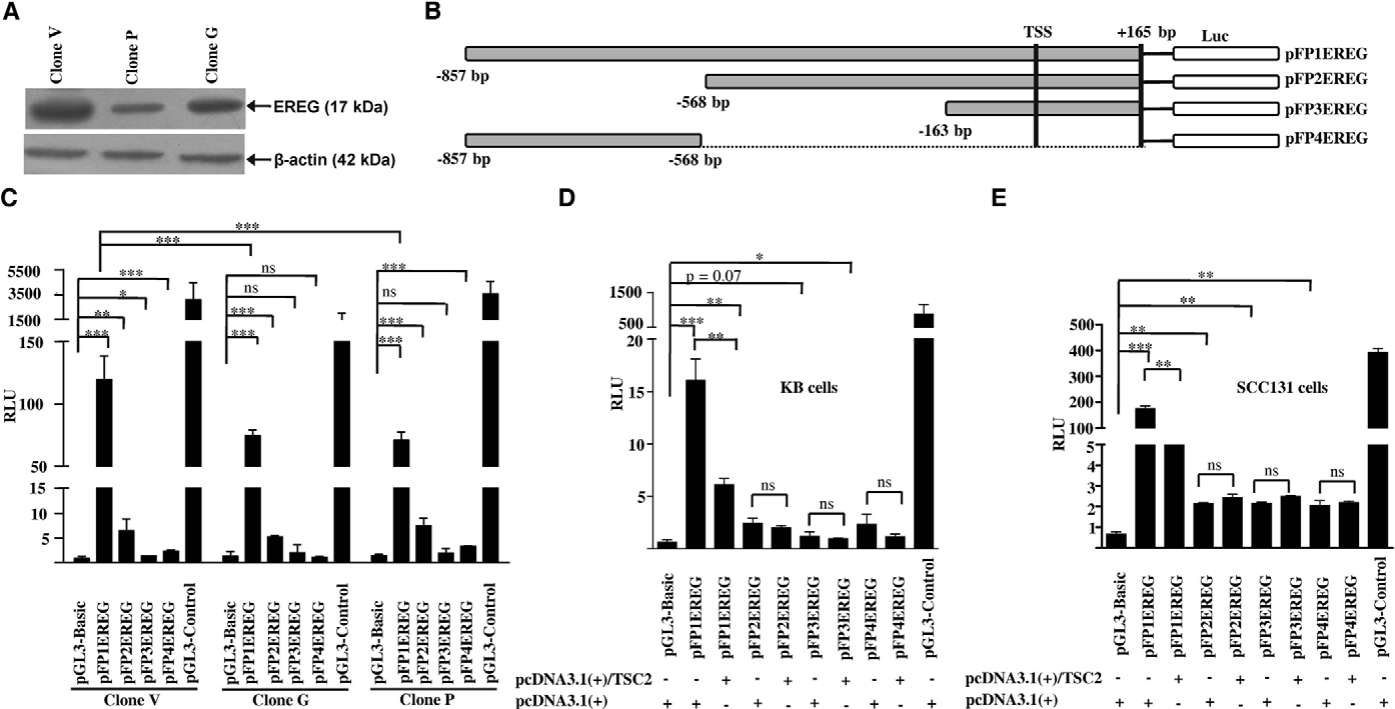
Downregulation of EREG in TSC2 overexpressing clones and dual reporter luciferase assay of *EREG* promoter deletion constructs. (**A**) Western blot analysis of EREG expression in stable clones. Note, overexpression of TSC2 in P and G clones leads to downregulation of EREG. (**B**) A diagrammatic representation of the promoter deletion constructs. TSS represents the transcription start site. (**C**) The promoter activity of deletion constructs in stable clones. (**D, E**) The promoter activity of deletion constructs in the presence (+) and absence (−) of a transiently transfected full-length TSC2 construct pcDNA3.1(+)/TSC2 in two different cell lines: KB and SCC131. The bars represent the mean ± SD of triplicate experiments performed twice. RLU represents relative luciferase unit. ns, data non-significant, *P < 0.05, **P < 0.01 and ***P < 0.001.

In order to validate if TSC2 transcriptionally represses the *EREG* promoter, we first analyzed its expression by western blotting. As expected, EREG was downregulated in clones P and G in comparison to clone V (Figure 5A). We then performed a dual luciferase reporter assay to confirm if indeed TSC2 transcriptionally regulates EREG expression using four *EREG* promoter deletion constructs harboring an upstream region from −857 bp to +165 bp (Figure 5B).

We adopted two experimental strategies to determine the effect of stable as well as transient overexpression of TSC2 on the *EREG* promoter. Deletion constructs were either transiently transfected in stable clones overexpressing TSC2 (clones P and G) and the vector clone V (Figure 5C) or were co-transfected with a full-length TSC2 construct pcDNA3.1(+)/TSC2 or the empty vector pcDNA3.1(+) in KB or SCC131 cells (Figure 5D and E). Under both conditions, the largest construct pFP1EREG displayed the highest promoter activity (Figure 5B, C, D and E). Further, the results demonstrated that the TSC2 overexpressing cells from stable clones G and P showed decreased luciferase reporter activity for the pFP1EREG construct relative to cells from the vector control clone V (Figure 5C). Co-transfection of TSC2 [pcDNA3.1(+)/TSC2] with the pFP1EREG construct in KB and SCC131 cells corroborated the above results (Figure 5D and E).

### TSC2 binds to the *EREG* promoter *in vivo*

To test whether TSC2 binds to the *EREG* promoter *in vivo* and regulates its expression, we performed the ChIP analysis, using an anti-TSC2 antibody. Each ChIP experiment had a total input DNA (INP lane), which served as a positive control for the PCR reaction, a pull-down with an anti-TSC2 antibody (+Ab lane) and a negative control without an antibody (−Ab lane).

**Figure 6.**
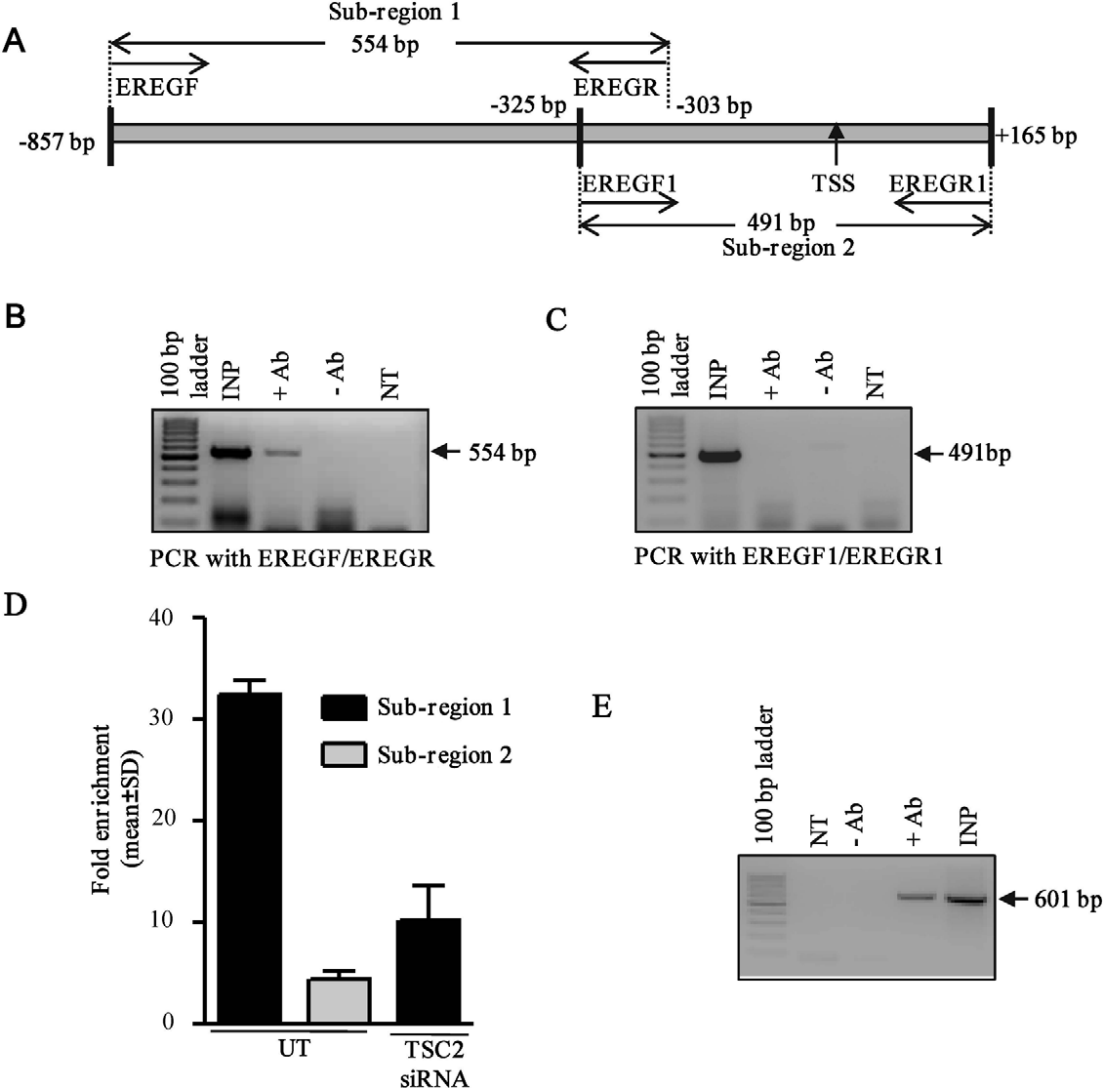
ChIP analysis to determine *in vivo* binding of TSC2 to the *EREG* promoter. (**A**) Diagrammatic representation of the sub-regions 1 and 2 of the promoter along with the primer pairs. (**B**) TSC2 binds to the promoter region from −857 bp to −303 bp. The INP lane represents the amplification from sheared chromatin as a template, and NT is no template control. (**C**) The promoter region from −325 bp to +165 bp was negative for TSC2 binding. (**D**) The relative quantity of promoter enriched by ChIP was quantified by qPCR and expressed as the fold enrichment of the promoter sub-regions 1 and 2 after normalization to a rabbit IgG. UT represents untransfected cells. TSC2 siRNA was used at a final concentration of 100 nM. Data represent the means of two independent experiments. (**E**) DNA binding of SP1 transcription factor to the P1 promoter of the *SLC22A18AS* gene as a surrogate positive control for the ChIP protocol. Note, the amplification in the +Ab lane with primers SP1F and SP1R.

Since TSC2 expression was able to repress the luciferase activity of the pFP1EREG fragment (Figure 5), it was used in the ChIP analysis. This fragment was divided in two overlapping sub-regions with two primer pairs. These primers spanned −857 bp to −303 bp (sub-region 1) and −325 bp to +165 bp (sub-region 2) of the *EREG* promoter (Figure 6A). In the PCR for the sub-region 1 (EREGF/EREGR primer set), an expected size PCR product of 554 bp was observed in the input and ChIP lanes with the anti-TSC2 antibody (Figure 6B, INP and +Ab lanes), suggesting binding of TSC2 to the *EREG* promoter in this region. On the other hand, an expected size PCR product of 491 bp was observed only in the input lane, whereas no amplification was observed in the ChIP lane with the anti-TSC2 antibody for the sub-region 2 (Figure 6C, INP and +Ab lanes), suggesting non-binding of TSC2 to this region. Since the −325 bp to +165 bp sub-region 2 of the *EREG* promoter did not amplify in the ChIP assay with the anti-TSC2 antibody, it served as a negative control. The enrichment of *EREG* promoter sub-regions was also quantified using qPCR. The results showed that the sub-region 1 of the promoter was specifically enriched compared to the sub-region 2 (Figure 6D). Further to validate the specific binding to the *EREG* promoter, TSC2 was knocked down in SCC131 cells and the ChIP assay was performed. The results showed the loss in enrichment of the sub-region 1 (Figure 6D). Overall, the above observations suggested that TSC2 binding to the *EREG* promoter is a specific event.

Since TSC2 has not been shown to bind to the DNA/promoter of any gene and therefore a positive control for DNA binding of TSC2 was unavailable, we wanted to see if our ChIP experiment was working by using binding of the SP1 transcription factor to the P1 promoter of the *SLC22A18AS* gene as a surrogate positive control. As expected, a PCR product of 601 bp was observed in both the input and ChIP lanes with an anti-SP1 antibody (Figure 6E), suggesting that our ChIP protocol was working.

### TSC2 binds to the *EREG* promoter between −352 bp and −303 bp region *in vitro*

**Figure 7.**
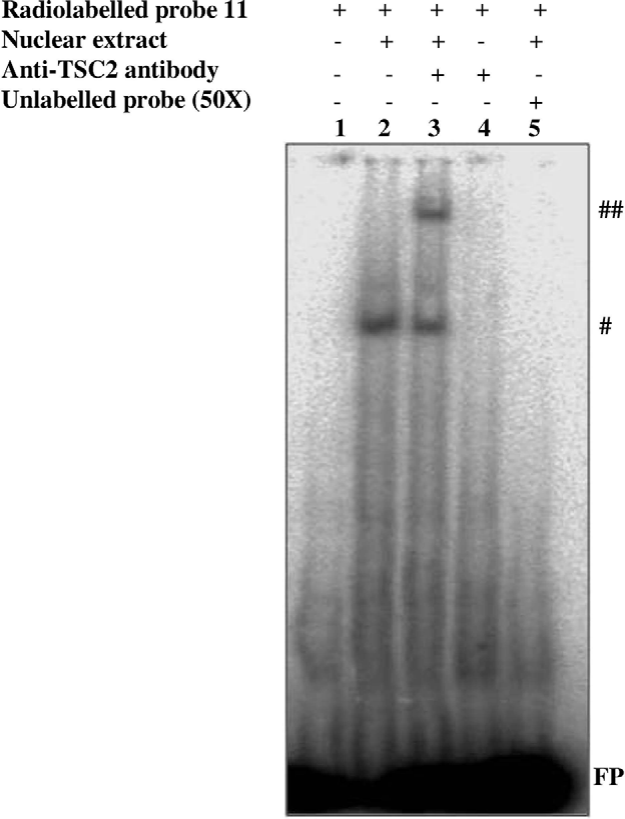
EMSA for determining the TSC2 binding region on the *EREG* promoter. The end-labeled probe 11, encompassing a region from −352 bp to −303 bp of the promoter, was incubated separately with the nuclear lysate from SCC131 cells in the presence (+) or absence (−) of the anti-TSC2 antibody. Note, the shift (#) and supershift (##) showing the binding of TSC2 to the promoter. FP represents the free probe.

In order to narrow the region of TSC2 binding on the *EREG* promoter and validate our ChIP data, we performed EMSA using 11 overlapping probes (probes 1 to 11) of ∼50 bp each (Supplementary Table S4), spanning the sub-region 1. Only the probe 11, spanning −352 bp to −303 bp, showed a supershift following preincubation of the nuclear extract from SCC131 cells with end-labeled probes and an anti-TSC2 antibody (Supplementary Figure S2). We repeated EMSA experiments with the probe 11, and the results showed that TSC2 indeed binds to the *EREG* promoter in this region (Figure 7). In order to further narrow the TSC2 binding region on the promoter, the probe 11 was split into six 15 bp overlapping sub-probes. However, we did not observe any binding of TSC2 to these sub-probes by EMSA (data not shown). This may be either due to disruption of TSC2 binding site(s) or the insufficient size of our sub-probes. Further, the ClustalW2 alignment of putative promoter sequences of 24 TSC2 regulated genes with the *EREG* probe 11 sequence suggested conservation of the 5’GCCTTG3’ motif in 11/24 promoters analyzed (Supplementary Figure S3). This motif was not present in the rest of the sub-region 1 (i.e. excluding probe 11 sequence) and sub-region 2 (data not shown), suggesting that it could be the putative TSC2 binding site.

**Figure 8.**
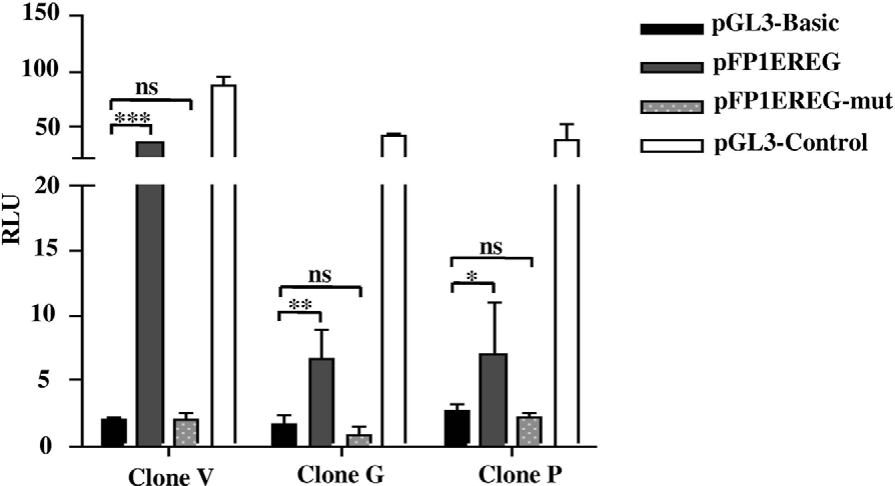
Deletion of the region harboring the TSC2 binding site inactivates the *EREG* promoter. Note, the loss of promoter activity (RLU) in the pFP1EREG-mut construct. ns, data not significant, *P < 0.05, **P < 0.01 and ***P < 0.001.

To validate the binding of TSC2 to the *EREG* promoter in a site-specific manner and test if the deletion of 50 bp from the pFP1EREG construct leads to the loss of TSC2 modulated luciferase expression, the construct pFP1EREG-mut having the deletion was transiently transfected in the vector clone V and TSC2 overexpressing clones P and G. The expected results were inconclusive as the deletion of the region led to the loss of promoter activity in the pFP1EREG-mut construct (Figure 8).

### Effect of TSC2 NLS deletion on EREG expression

**Figure 9.**
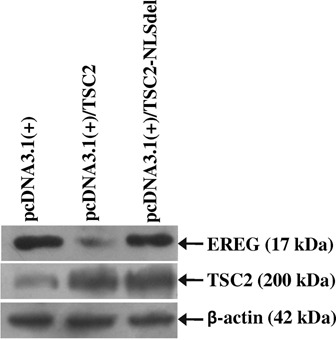
Effect of TSC2 NLS deletion on EREG expression. Note, the deletion of NLS in TSC2 abolishes the downregulation of EREG expression.

In order to determine the effect of TSC2 NLS deletion on the expression of EREG, we generated the pcDNA3.1(+)/TSC2-NLSdel construct harboring the NLS deletion, transfected in SCC131 cells and assessed the levels of TSC2 and EREG by the western blot analysis. The results showed the overexpression of TSC2 in cells transfected with the full-length TSC2 wild-type (pcDNA3.1(+)/TSC2) and pcDNA3.1(+)/TSC2-NLSdel constructs as compared to the vector (pcDNA3.1(+)) transfected cells (Figure 9). As expected, the level of EREG was reduced in cells transfected with the wild-type TSC2 construct as compared to the vector transfected cells (Figure 9). However, the level of EREG in pcDNA3.1(+)/TSC2-NLSdel transfected cells was more than the wild-type TSC2 construct transfected cells and similar to the vector control (Figure 9), suggesting that the deletion of NLS indeed abolishes the TSC2-mediated EREG repression.

### Non-canonical regulation of EREG by TSC2 and the effect of TSC2 overexpression on mTORC1 and mTORC2

**Figure 10.**
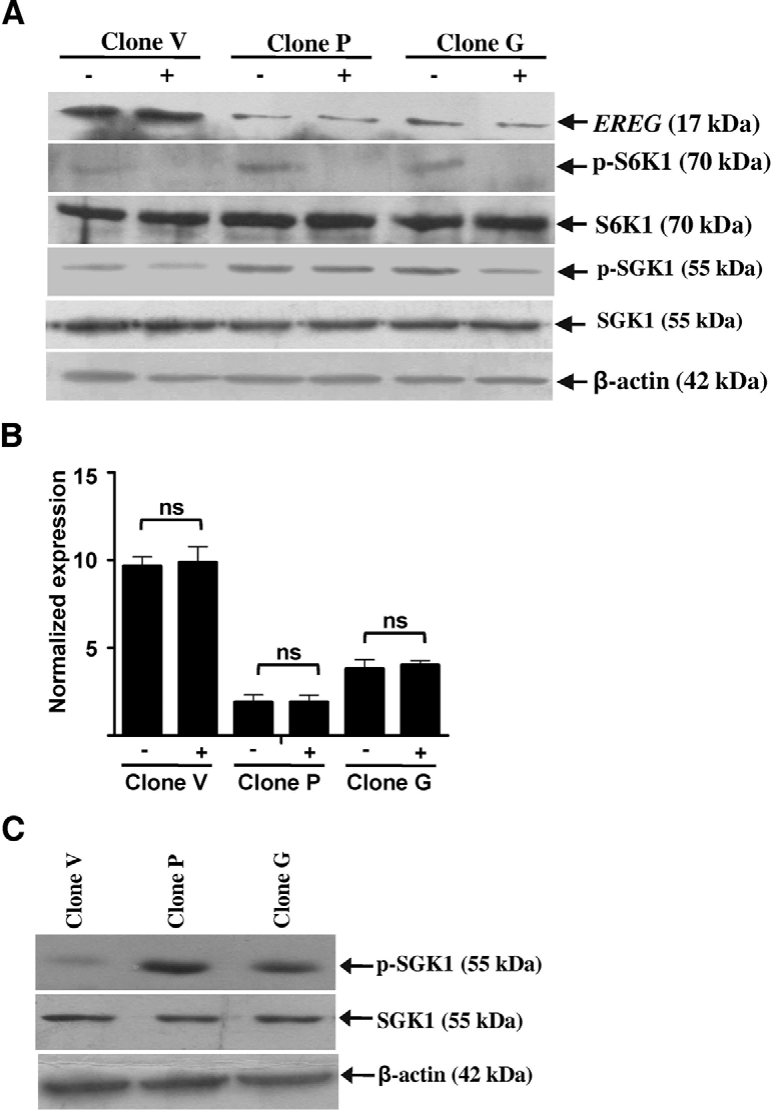
Non-canonical regulation of EREG by TSC2 and the effect of TSC2 overexpression on mTORC2. (**A**) Western blot analysis to assess the levels of EREG, p-S6K1, S6K1, p-SGK1 and SGK1 following the treatment of cells from stable clones with 100 nM rapamycin (+) or vehicle control DMSO (−). β-actin was used as a loading control. (**B**) qRT-PCR analysis of *EREG* following the treatment of cells from stable clones with 100 nM rapamycin (+) or DMSO (−). *RPL35A* was used as a normalizing control. ns, data not significant. (**C**) Western blot analysis of cells from stable clones to assess the levels of SGK1 and p-SGK1. β-actin was used as a loading control.

In order to determine the effect of TSC2 overexpression on mTORC1 and to differentiate between the canonical and non-canonical functions of TSC2, the level of phosphorylated S6K1 (p-S6K1) was analyzed as a readout for mTORC1 activity following treatment of the cells with rapamycin. The basal level of p-S6K1 in clones P and G was higher than the vector clone V (Figure 10A). Upon 24 h of the rapamycin treatment, the level of p-S6K1 decreased in the vector clone V and the TSC2 overexpressing clones P and G, confirming the inhibition of mTORC1 (Figure 10A). We also assessed the level of p-SGK1 as a readout for mTORC2 activity following 24 h of rapamycin treatment. The results showed a partial inhibition of mTORC2 activity, as shown by the modest decrease in the level of p-SGK1 in the vector clone V and the TSC2 overexpressing clones P and G (Figure 10A). As observed by western blot and qRT-PCR analyses, the expression of EREG did not change both at the protein and transcript levels post rapamycin treatment in TSC2 overexpressing clones as well as in the vector clone V (Figure 10A and B). The unchanged *EREG* level suggested that its transcriptional regulation by TSC2 is insensitive to rapamycin. The results also showed an increased level of p-SGK1 in TSC2 overexpressing clones as compared to the vector control clone V (Figure 10C), suggesting that an increased level of TSC2 leads to increased activity of mTORC2.

### Effect of TSC1 knockdown on TSC2 and EREG expression

**Figure 11.**
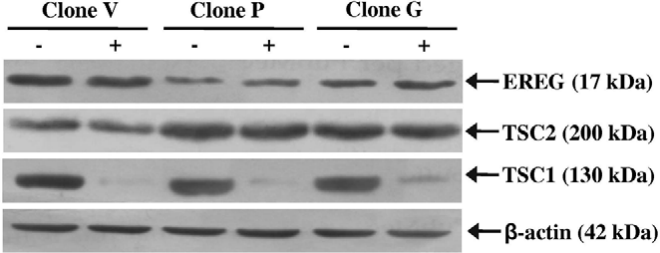
Effect of TSC1 knockdown on the levels of TSC2 and EREG. Western blot analysis of stable clones transfected with 100 nM of TSC1 siRNA (+) or scrambled oligos (−). β-actin was used as a loading control.

To determine the effect of TSC1 knockdown on the levels of TSC2 and EREG, we transfected the TSC1 siRNA in clones V, P and G. The results showed a drastic decrease in the level of TSC1 in TSC1 siRNA transfected cells (+) as compared to scrambled oligo (−) transfected cells (Figure 11). However, we did not observe any change in the levels of TSC2 and EREG (Figure 11), suggesting that TSC1 has no effect on the expression of EREG.

## DISCUSSION

Rosner *et al.* (20) have studied the gene expression responses to transient overexpression of TSC1 and TSC2 in HeLa cells. They not only provided new insights into the cellular roles of TSC proteins, but also documented that both of these proteins regulate non-overlapping differential gene expression (20). Although their study revealed novel regulatory targets of TSC proteins, only 2400 genes were used in the microarray analysis (20), in contrast to our study of 19 596 genes, and thus a vast majority of the genes regulated by TSC2 remained to be discovered. Additionally, Li *et al.* (21) performed a whole-genome microarray analysis on fibroblast-like cells grown from angiofibromas, periungual fibromas and normal fibroblasts of TSC patients with allelic deletion of *TSC2*. The *EREG* gene was found to show the greatest mean elevation in expression in angiofibromas and periungual fibromas (21), thus corroborating with our observation that it is the most downregulated gene upon TSC2 overexpression in both the clones (Supplementary Table S7). This concordance with previously published data especially from TSC patients (21) further strengthened the validity of TSC2 regulation of *EREG*. Moreover, EREG was induced in TSC skin tumor cells by recombinant Epidermal growth factor (EGF) (21). However, inhibitors to Epidermal growth factor receptor (EGFR) and MAP kinse-ERK kinase (MEK) (a downstream molecule in the EGFR pathway) did not change the elevated level of EREG in TSC skin tumor (21). Thus, the elevated EREG expression in TSC tumor cells was not accounted for by the EGFR signaling (21). On the whole, the involvement of TSC2 regulation of EREG was reinforced by various studies as discussed above (17,21). Hengstschläger *et al.* (22) have performed proteomic analysis of TSC1 and TSC2 overexpressing HeLa cells and observed deregulated levels of 21 and 22 proteins upon TSC1 and TSC2 overexpression, respectively. However, the altered level of EREG was not detected (22). The reason Rosner *et al.* (20) were not able to find the downregulation of *EREG* in their microarray analysis is due to the absence of its probe on the Micromax Human cDNA System I-Direct array (cat. no. MPS102, Perkin-Elmer Life Sciences, Hopkinton, MA, USA) used by them. Interestingly, two of the genes, *MAP1B* (Supplementary Table S6) and *CCND1* (Supplementary Table S10), found in our study were also identified as TSC2 regulated genes by Sen *et al.* (12).

Our results showed that the TSC2-mediated transcriptional repression of EREG was insensitive to rapamycin, suggesting that it was independent of mTORC1 and thus non-canonical in nature. Interestingly, we also observed an increased level of p-S6K1 in TSC2 overexpressing clones compared to the vector clone V (Figure 10A). Although a counterintuitive finding, an increased level of p-S6K1 has been observed previously in TSC2 overexpressing mouse embryonic fibroblast and glioblastoma cells (7,23). We also observed a partial inhibition of mTORC2 activity upon 24 h of the rapamycin treatment as reported earlier by Sarbassov *et al.* (24) in U87, SKW3 and UACC-903 cells. It has been reported that an increased TSC2 level leads to activation of mTORC2 which in turn activates AKT and leads to an increased level of p-S6K1 (7,23).

TSC2 acts as a tumor suppressor in a variety of cancers (e.g. oral, lung and ovarian cancers) (25,26), and the upregulation of EREG has been documented in a variety of cancers (e.g. colorectal, lung, pancreatic, oral and ovarian cancers) and in TSC patients (21,27–31). Moreover, EREG has been suggested to be a plausible ‘hamartic’ factor in TSC (21). EREG, a secreted protein, is capable of activating EGFR (21,32), which is a potent survival signal and activates the MAPK as well as the PI3K-AKT-mTOR pathways. Thus, we suggest that monoclonal antibodies or inhibitors against EREG may have therapeutic value in a variety of malignancies as well as in TSC patients. Currently, mTORC1 is targeted in many clinical trials for the treatment of TSC (http://clinicaltrials.gov/ct2/results?term=Tuberous+sclerosis&Search=Search). We believe that the inhibition of EREG may have advantage over inhibition of mTORC1 as this strategy may inhibit both the downstream pathways.

As stated above, TSC2 modulates the transcription mediated by members of the steroid receptor superfamily and ERα (10,11). Thus, the role of TSC2 in transcription has been documented before. However, this is the first study which showed the direct nuclear function of TSC2, which binds to the promoter of *EREG* between −857 bp and +165 bp and represses its level. This promoter region has previously been shown to be transcriptionally active (17). Further, EMSA results confirmed the binding of TSC2 to the *EREG* promoter between −352 bp and −303 bp. We were unable to narrow the TSC2 binding region on the *EREG* promoter further due to the unavailability of a full-length purified TSC2 protein. However, using bioinformatics approaches, we were able to identify a conserved 5’GCCTTG3’ motif in the promoters of 11/24 TSC2 regulated genes (Supplementary Figure S3), suggesting that this could be the putative TSC2 binding site. We hope that the future study with a full-length purified TSC2 protein will validate if this motif is indeed the TSC2 binding site on the *EREG* promoter and the promoters of other TSC2 regulated genes observed in this study.

## SUPPLEMENTARY DATA

Supplementary Data are available at NAR Online.

SUPPLEMENTARY DATA
