# Supplementary material for: Evidence that TSC2 acts as a transcription factor and binds to and represses the promoter of Epiregulin
Source: Nucleic Acids Res. 2014 Apr 19;42(10):6243–55. doi: 10.1093/nar/gku278 (PMC4041451; doi:10.1093/nar/gku278)
Supplement: SUPPLEMENTARY DATA [file supp_42_10_6243__index.html]

Evidence that TSC2 acts as a transcription factor and binds to and represses the promoter of Epiregulin — Evidence that TSC2 acts as a transcription factor and binds to and represses the promoter of Epiregulin — SUPPLEMENTARY DATA 

# Evidence that TSC2 acts as a transcription factor and binds to and represses the promoter of Epiregulin

## SUPPLEMENTARY DATA

**Files in this Data Supplement:**

- SUPPLEMENTARY DATA
- SUPPLEMENTARY DATA
- SUPPLEMENTARY DATA
- SUPPLEMENTARY DATA
