# Supplementary material for: Evidence that TSC2 acts as a transcription factor and binds to and represses the promoter of Epiregulin
Source: Nucleic Acids Res. 2014 Apr 19;42(10):6243–55. doi: 10.1093/nar/gku278 (PMC4041451; doi:10.1093/nar/gku278)
Supplement: SUPPLEMENTARY DATA [file supp_gku278_nar-03278-v-2013-File013.pdf]

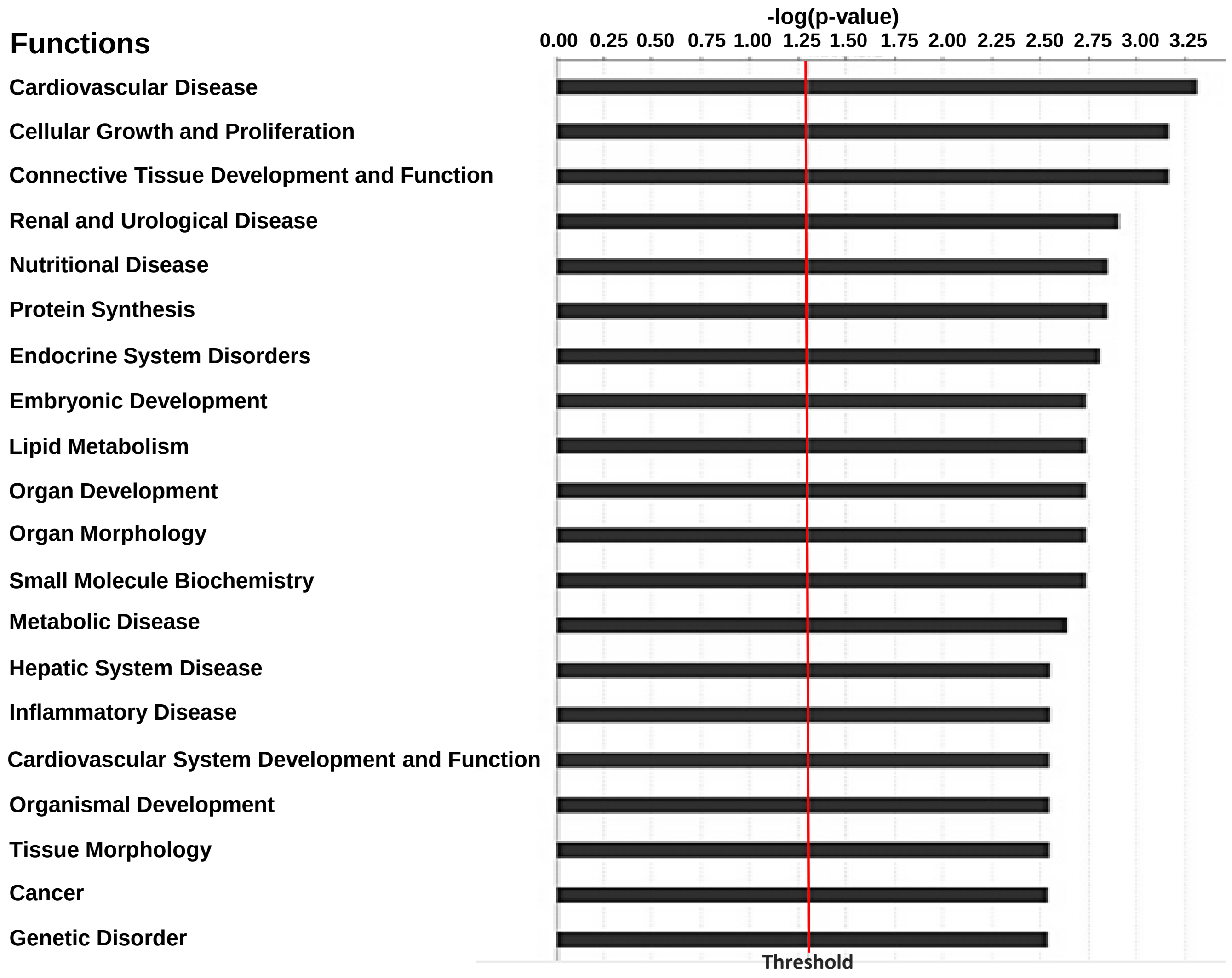

Supplementary Figure S1. Functional annotation of the genes regulated by TSC2. The top 20 functional categories picked up by the IPA software. These functional categories were significantly associated with the microarray data. A $p<0.05$ was considered to be significant by the IPA software as seen from the threshold marking (red line).

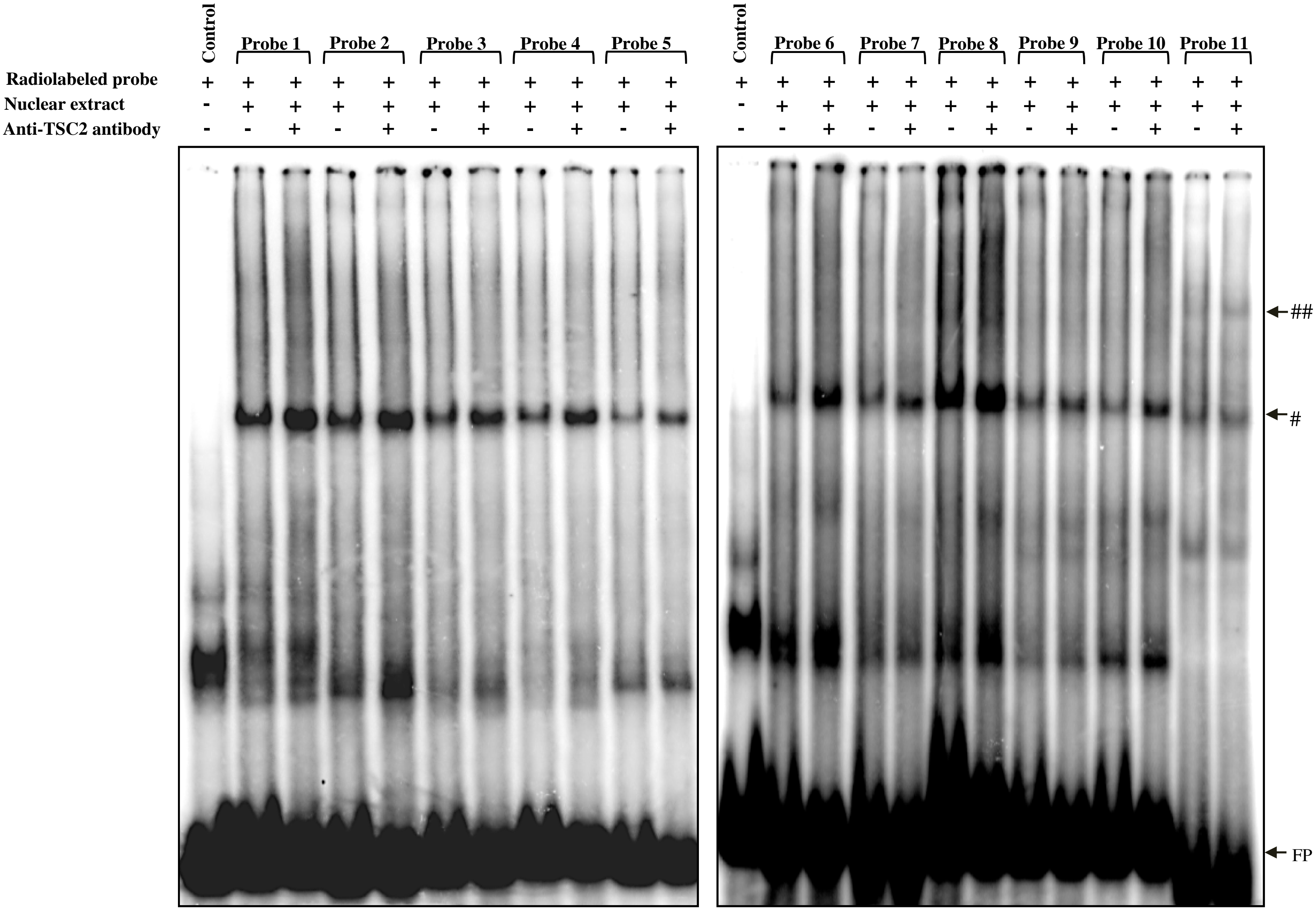

Supplementary Figure S2. Determination of the TSC2 binding region on the *EREG* promoter by EMSA. The end-labeled probes (probes 1-11), together encompassing a region from -857 bp to -303 bp of the *EREG* promoter, were incubated separately with the nuclear lysate from SCC131 cells in the presence or absence of the anti-TSC2 antibody. Note the shift (#) and supershift (##) in the probe 11, showing the binding of TSC2 to the promoter. The control lane represents the end-labeled probe 1 in the absence of the nuclear extract and the anti-TSC2 antibody.

AK-Supplementary Figure S3

```
Probe11         GCCT-TGG-------------------------------CTAAAAGACT--GATTCAAGT 26
PPP1R14C        GCCTCTGGGGGCTGGACCGA-GAGGGTCCTCGGCGGCTTCTTTAAGGCTGCGGTCCCCGC 589
                **** ***                               **::***.**  *.* *..*

Probe11         TATGTC-----------------------------CG-----------TCACCCTGAGCA 46
PPP1R14C        CCCGTCGTCCT-CCCCGCCCTCCTCCCTCTCGCCGCGGCTCCTCCCGCCCTCCCAGAGCA 648
                 . ***                             **            *:***:*****

Probe11         AC-C-------------------------------------------------------- 49
PPP1R14C        GC-CGGGCGGCTGGGCGCGCGCGGCGCAGAGCAGGTGCCGGGGAGCCCTTCGC-ATGCGG 706
                .* *


Probe11         -----------------------------------------GCCTTGGCT---------- 9
SERPINB13       TAATGTATGTGTTCATCAGGCTATACACC-TTTGGTTCCCAGCGTTGGCTGTACATTGTA 530
                                                         ** ******

Probe11         ----------------AAAA--------GACTGATT------------------------ 21
SERPINB13       ATCACCTGCGGTGATAAAAA-TTCTGATGCCTGGTTTCCACTCGAGAGATGCTGACTCAG 589
                                ****        *.***.**

Probe11         --------------------------------------------CAAG------------ 25
SERPINB13       TTGGTCTGGGG-TGGGGCTTGGGCATAGATTTTTTTAAAGTTCCAAGGAGATTTTAAGG 648
                                                            ****

Probe11         ------------------------------------------------------------
SERPINB13       TG-CACGCAAGGTGGAAAACCACTGCTGAAGCAGATGTGGAGAACTATAAATT-AAGGAT 706

Probe11         ------------------TTATG-------------------------TCCGTCAC---- 38
SERPINB13       CCCAGCTACTTAATTGACTTATGCTTCCTAGTTCGTTGCCCAGCCACCACCGTCTCTCCA 766
                                  *****                         :*****:*

Probe11         ----CCTGAG--------------------CAA-------CC------------------ 49
SERPINB13       AAAACCCGAGGTAAGTTCTTATTTTGGCTCCAATTTAAGACCTCTGTATTTTGAGAACAA 826
                    ** ***                    ***       **


Probe11         ------------------------------------------------------GCCTTG 6
MAPK4           GA-GCTGGAGCAGCGAGCCGGGCTGTCGGGGCGACCGCGGGAGCTCGCCGTGC-GCCGTG 706
                                                                      *** **

Probe11         GCT-----------------------------------------AAAAG----------- 14
MAPK4           GCTGGGACCGGCCTGGCCGAGCGCGCCGGCGCCGCGGCCGCAGACAAAGGGCGGCTCGCG 766
                ***                                         .****

Probe11         -----------------------ACTGATTC---AAGT---------------------- 26
MAPK4           CCCGGGCCGCCACGCTCTCGGGCTCTGCCTCGGTAAGTGGCTCCCCTCCGCTGGCTTTCT 826
                                       :***. **   ****

Probe11         --------------------------TATG------TCCG-------------------- 34
MAPK4           CCTCCCGCCGCCTGCGCCTCTCGGAGTTCGGCGGGCTCCGGAGAAGCGGGGAAGAGATGA 886
                                          *: *      ****

Probe11         ----TCACC-------CTG------------------------AGCAACC---------- 49
```

```
MAPK4           GACTTCCCCGCCCGCACTGCCTCCCCACCTTACCCTAACAATAAGCCCCCCAGGCCAAGC 946
                    **.**       ***                           ***..**


Probe11         --------------GCCTTGGCTAAAAGACTGATT-----------CAAG---------- 25
IL18            TTCGACTTCCA-TTGCCCTAGGAAAGAGCCTGTTTGAAGGCGGGCCCAAGGAGTGCCGAC 648
                              *** *.* :**.**.***:**           ****

Probe11         ------------------------------TTATGTCC--------------------- 33
IL18            AG-CAGTCTCCTCCCTCCACCTTCTTCCTCATTCTCTCCCCAGCTTGCTGAGC-CCTTTG 706
                               **.* ***

Probe11         -------------------------------------GTCACCCTGAGCAA----CC--- 49
IL18            CTCCCCTGGCGACTGCCTGGACAGTCAGCAAGGAATTGTCTCCCAGTGCATTTTGCCCTC 766
                                                     ***:***:*:***:    **


Probe11         -----------------------------------------------------------G 1
ADIPOQ          ATACCAGAGGGTAAGAGCAATTCTGTGAAGTTCCAGGCTGGGTGGGGGATGCATGCATAG 826
                                                                           *

Probe11         CCT-TGGCT-------AAAAGACT-------------------------GATTCAAGT- 26
ADIPOQ          CCTCTGGCTGGGATCACCCAGGCTCTCCCGTCCGTAGTAGTGTGGGAGTGGATACAGGTG 886
                *** *****       ...**.**                          ***:**.**

Probe11         -----------------------------------TATG---------------TCCG-T 35
ADIPOQ          GATACTCTGGTCAGAGCAGCACTGGTGGAGGCAGATATGCACTGGGCTTCTTCCTCCGTT 946
                                                   ****               **** *

Probe11         CACCCTGAGCAACC---------------------------------------- 49
ADIPOQ          CTCCCACAGCCCCAAGAGAGAAAGGGTTATTTCAGACATTCCTTCTAAGATGCA 1000
                *:***: ***..*.


Probe11         -----GCCTTGGCTAAAAGACTGATT---------CAAGTTAT-----------GTC--- 32
MAP1B           ACCTCGCCTAGCCTGTCGCACCGCTTCCTTGACAGCAAGTTCTACTTGCTGGTGGTCGTC 886
                     ****:* **.:.. ** *.**         ******.*          ***

Probe11         --------CGT------------------------------------------------- 35
MAP1B           GGCGAGATCGTGACCGAGGAGCACCTGCGGCGTGCCATCGGCAACATCGAGCTCGGTAAG 946
                        ***

Probe11         ----------CACCCTGAG------------------------CAACC----- 49
MAP1B           TGGCCCCGCGCCCCCAGAGACGCGCGCTGGGAGACGCGCAAACACGACCCCACC 1000
                          *.***:***                          *.***


Probe11         ------------------------------------------------------GCCTTG 6
```

```
ST6GAL1         ATTTCACCATGTTGGCCAGGATGGTCTCGATCTCCTGACCTCGTGATCCA-CCTGCCTTG 59
                                                      ******

Probe11         G---CTAAAAG-ACTG---ATTCAAGT---------------------TATGT------- 31
ST6GAL1         GCCTCCCAAAGTGCTGGGAATACAGGCGTGAGCCATCATGC-CTGGACTATGTGTTGGTT 118
                *   * .**** .***   **:**.*                      *****

Probe11         ------------------------------------------------------------
ST6GAL1         TCCATGCATGTTTCCTGGCGCATAGCCCCATA-GCCCTTGCTACAGTCTTGCTAGAATGC 177

Probe11         ---------------------------------------------------CCG-----T 35
ST6GAL1         TGGGCTTTTTAGACTTCAGGGAC-AGGCCCTAAGAAACAGTCTCTCTCCTGCCGTCCTTT 236
                                                                   ***     *

Probe11         CACC-------------------------------------CTGAG-------CAACC-- 49
ST6GAL1         CACCTGCCCCAAGG-CAAGGTTTTTATGTTTTCTGGCCTGACTGTGGGCCTTACAACCCT 295
                ****                                     ***:*       *****



Probe11         ----------------------------------GCCTTGG-----------------CT 9
APOC1           AATCCACAAGCAGACAGCCCGCGCCACACCCTGGGCCTGGT-GGTTAAAGATTGACCCCT 118
                                                  **** *                  **

Probe11         ---------------------------------------------AAAAG-ACTG----- 18
APOC1           GACCTAATCCGTTAGGTTATCTATAGATTACA-GACATTGTATAGAAAAGCACTGTGAAA 177
                                                             ***** ****

Probe11         ATTCAAGTTATG------TCCGT----------------------CACCCTG---AGCAA 47
APOC1           ATCCCTATTCTGTTTTGTTCCGA-TCTAATTACCGGTGCATGCAGCCCCCAGTCACGCAT 236
                ** *.:.**.**      ****:                      *.***:*   .***:

Probe11         CC---------------------------------------------------------- 49
APOC1           CCCCTGCTTGTTCA-ATCGATCACGACCCTCTCACGTGCACCCACTTAGAGTTGTGAGCC 295
                **



Probe11         -----------------------------------G---------CCT--TGG-CTAAAA 13
PCDH9           ACCTATCCTTTGAAAATGTGAGTACCAAGATAAGTGTTCAATTACCCTAATGGTCTTCAA 886
                                                   *         ***  *** **:.**

Probe11         GACTG------------ATTCAAG-----------------TTATG-------------- 30
PCDH9           AACTATTCTTTAGGGAAATTCATGGAGAAAGTTCATTGACTTTCTGGTCAAGATTCATGC 946
                .***.            *****:*                 **.**

Probe11         ----TCCGTCA-------CCCTG---AGCAACC--------------------- 49
PCDH9           AAATTTCATCAGTGAAATCCCAAGTCAGAAACTGTGCTGGTAGCACTAGTTAGT 1000
                    * *.***       ***:.   **.***



Probe11         ---------------GCCTTGGCT------------------------------------ 9
ASMT            CCAGAGGCTCCGGAAGCCACGGCTGGATTGGAGACAAGATGGGATCCTCAGAGGACCAGG 826
```

```
                               ***: ****

Probe11         -------------AAAAGACT---------GATTCAAG---------------------T 26
ASMT            CCTATCGCCTCCTTAATGACTACGCCAACGGCTTCATGGTGTCCCAGGTAGGATACGCTC 886
                             :**:****         *.****:*

Probe11         TATG-------------------TCCG----TCACCCT---------------------- 41
ASMT            TGTGGGACAAGGGGGAATAGACTTCCGTTCATCACACTCACGTTCCATTGTTGTGTCAGT 946
                *.**                   ****    ****.**

Probe11         -------------------------------GAG---------CAACC------ 49
ASMT            CGAGAAAAATGATGAGTCTCCATCATTTAGGAGGTTTATTTTCCACCGTGAAA 1000
                                               ***         *.***



Probe11         ------------G------CCTTGGCTAA-AAGACTG----------------------- 18
FOXF1           CCCGCGTCGTCCGGCCCGTCCAAGGCCAAGAAGACCAACGCCGGCATCCGGCGCCCGGAG 766
                            *      **::*** ** ***** .

Probe11         ----------ATTCA--------------AGTTATG----TCCG----TCACCC------ 40
FOXF1           AAGCCGCCCTATTCCTACATCGCGCTCATCGTCATGGCCATCCAGAGTTCACCCACCAAG 826
                          ****.              .** ***    ***.    ******

Probe11         ----------TGAGC------AACC----------------------------------- 49
FOXF1           CGCCTGACGCTGAGCGAGATCTACCAGTTCCTGCAGAGCCGCTTCCCCTTCTTCCGGGGC 886



Probe11         -----------------------------------------------GCCTT-------- 5
HIVEP3          AAGCACCAATAAATGAAATCCTTATCACTGTG-TGCAAGCCTACTGTGCCTTTAGGCATG 177
                                                               *****

Probe11         -----GGCT--------------------------------------------------- 9
HIVEP3          GGACTGGCTTCCTGCGCTGGGAG-TGACAGCTATTCCGAAGTGATAATTGGTGTACTGTG 236
                     ****

Probe11         -----------------------------AAAAGACTG---------------------- 18
HIVEP3          TCTTTAAAAGGTTA-AACCCCCTTTCAAAAAAGGTCTGGACTCCTAGTGGAAAAGACAAG 295
                                             ***.*:***

Probe11         ------------------------------------------------------------
HIVEP3          CGAGG-CATGGGTTGGCTCTGGATTCAGAAAACAGCTGCTGATTAGATTCTGTGTC-TGC 353

Probe11         -----------------------------------ATTCAAG------------------ 25
HIVEP3          AGGCTGAATTTATTCCGTGTTTTTCAGCAGCACGAATTCAAGAGGGA-TGCTTCTGTTTG 412
                                                   *******

Probe11         ---TTATGTCCGT--------------CA---------------------------CCCT 41
HIVEP3          GGTTTCTGATCGTGATTTATATTTATTCAGAGGAATAA-CAGAGGGTGGATCTGTTCCCG 471
                   **.**: ***              **                           ***

Probe11         GAGC------------AACC---------------------------------------- 49
HIVEP3          GCGCGAGCATGCTCACAACCAGCCGACTC-TCCCATTATCCAGCTGCCTAGTTTGGTGCT 530
                *.**            ****
```

```
Probe11          -----------------------GCCTTGG-----CTAAAAGACT----------GATTC 22
UNC13A           GGGCGCGCTGG-GGTCAGCGCCGGCCTCGGGCGTCCTCAGGGGCTGCGGAGAAGAGCTCC 648
                                        **** **     **.*..*.**          *.* *

Probe11          AA----------G----------------------TTATG-------------------- 30
UNC13A           AA-GCACGGGGCGGAGGACCGGACACAGAACATCATCATGGTGCTCAAGGACC-GCATGA 706
                 **          *                      * ***

Probe11          ---TCCG-----------------------------TCACCCTG--------AGCA---A 47
UNC13A           AGATCCGGGAGCGCAACAAGCCCGAGATCTTCGAGCTCATCCAGGAGATCTTCGCGGTGA 766
                    ****                             *** **:*        .**.   *

Probe11          CC---------------------------------------------------------- 49
UNC13A           CCAAGACGGCGCACACGCAGCAGATGAAGGCGGTCAAGCAGAGCGTGCTGGACGGCACGT 826
                 **


Probe11          -------------------------------------------G---------------- 1
COL13A1          AGCGCGGCAGA-GGGGCTCCCGGGCCGGCGGGCTGGGGCCGCCGGACCTGCTCACCACGC 648
                                                            *

Probe11          --------CCTTGGC----------TAAAAGACTGATTCAAG------------------ 25
COL13A1          CC-CCCACCCTGGGCGAGTTTGCATAAATAAACAAATCCAAGCCGTCGGCCCC-GCGGGA 706
                         *** ***          :**:*.**:.** ****

Probe11          -----------------------------------------------------TTA---T 29
COL13A1          GGTGGAGGAGGAGTGACGAGGAGGAGGAGGAGGGAGGGGGGAGGGAGAGATTAGTTT 766
                                                                      ***   *

Probe11          G--------------------------TCCGT-------CA------------------- 37
COL13A1          GGGAAGTAGCACGGATTGCTCATCCGATCCGTGCCGCCGCAGGGAGTGTGTCAAGTTACA 826
                 *                          *****       **

Probe11          -----------------CCCTGAG--------CAACC----------------------- 49
COL13A1          GAGGCGCCGGAATCGGCCCCTGCGCTCCTCGCCAGCCGCCACGACCCACCTCTGCCCATG 886
                                  *****.*        **.**


Probe11          -----------------------------------------------------------G 1
NUDT11           TCCCCGGCGGCGGCAGCGGCAGCGGCGGCCCACACAGCAGCGAGAGGCGAGAGGAGGCTG 826
                                                                            *

Probe11          CCTT-----GGCT-------------------------AAAAGAC--------------- 16
NUDT11           CCTCGAGGAGGCTGCCTCGAGGATGAAGTGCAAACCCAACCAGACGCGGACCTACGACCC 886
                 ***      ****                         *..****

Probe11          ----TGATTCAAGTTATG-----------------TCCG------TCAC----------- 38
NUDT11           CGAGGGGTTCAAGAAGCGGGCGGCGTGCCTGTGCTTCCGGAGCGAACGCGAGGACGAGGT 946
                      *.******::. *                 ****      :*.*

Probe11          CCTG---------AGCAACC------------------------------------ 49
NUDT11           CCTGTTAGTGAGTAGCAGCCGGTACCCGGACCGCTGGATCGTGCCGGGCGGGGG 1000
```

AK-Supplementary Figure S3

```
                ****         ****.**





Probe11         -----------------------------------------------------GCCT--- 4
SLC6A12         TTCCGAATGTGCTCCAGGCAGCACCATTT-GCCATCCTGCTTCTAACGCAAACCCCTGAC 530
                                                      ***

Probe11         -----------------TGG-------------CTAAAAGACTGATTCAAG--------- 25
SLC6A12         TTCATGGATGAGGAACCTGG-AGACCAAAGAGACAAAGGGACTTTTTCAAGTTCACATGG 589
                                 ***             *:**..**** :******

Probe11         ---------------------------------------TTATGTCCGT----------- 35
SLC6A12         GGACCCCCTTC-TTGGGGGCCAGAGATATGACTAAAACCTTATCTCCTTGTGCTCAGGCC 648
                                                       **** *** *

Probe11         -----------------CACCCTG--------AGCAA--------------CC------- 49
SLC6A12         AG-TGTCTTCCCATTAACCCCCTGCCTTAGTTAACAAGTGTGTATGGATTGCC-TTCCAT 706
                                 *.*****        *.***              **




Probe11         ---------------------GCCT-------------------TGG----CTAAAAG-- 14
SEMA6A          AAGGAGAGAGCGCAGCCCGCGGCCCGGAGAGATCCCCTCGATAATGGATTACTAAATGGG 886
                                     ***                    ***    *****:*

Probe11         --ACTG---ATTCAAGTTA-------------------TGTCCGTC-------------- 36
SEMA6A          ATACACGCTGTACCAGTTCGCTCCGAGCCCCGGCCGCCTGTCCGTCGATGCACCGAAAAG 946
                  **:      .*:*.****.                   ********

Probe11         ----ACCCTGAGCAACC------------------------------------- 49
SEMA6A          GTACGCCCCGGGCGCCCGGGCGCGCCCCACCTCCCGGGCAGCTGGACTCCAGCC 1000
                    .*** *.**..**




Probe11         ---------G-------CCTTGG------CTA---------------------------- 10
TNFSF15         AGCTCCTCTGTCTTCATCCTTGG-ATGACCTAGGACAAGTGCCTTCACCTTTTAGAACTT 236
                         *       ******      ***

Probe11         ------------------------------------------------------------
TNFSF15         TGCTATTCCCATCT-GTACACTGAGAATGAATTTCTATGAGGTTCAAAATGACAGAGGGC 295

Probe11         ----------------AAAGA-CTGATTCAA--GTTATGTCCG------------------ 34
TNFSF15         TAGGC-AGCACTTTGCAAAGAGCTGATGCAAATGTGATTTCCGTTTCCCAATCTGC-AAA 353
                                ***** ***** ***  ** ** ****

Probe11         ------------------------------------------TCACC----CTG------ 42
TNFSF15         CCACACACCTCTCATCCCTCACTTTCACTTCCAGAAGCACACTCCCC-AGACTGGCACAG 412
                                                          **.**    ***
```

```
Probe11         ------AGCAACC----------------------------------------------- 49
TNFSF15         CTGGTAAGCCTCCAGTGCAGGGAACAGGTATATTTTAA-TTTGCCCCACCCACTGACTTG 471
                      ***.:**


Probe11         -------------------------------------------------GCCTTGG---- 7
CDC42EP5        TAGACTCCCACCTGGGCTTCCCA-CCTTCTTCCCACTGAGCTGCATCCTGCCTTGGGACT 236
                                                                 *******

Probe11         -----------------------------------------CTAAAAGACTG-------- 18
CDC42EP5        CTGCTCTCCCCCTC-CGCTCTCTCTCCCTGCCCGCACCGCCCTGAAGGAATTGCCCTAAC 295
                                                         **.**.**.*

Probe11         ATTCA--------------------------------------------AGTTATG---- 30
CDC42EP5        ACTCA-AACCTCACTCTCTTGTCATCAATAGACACTGGTGCTTAGAATGAGATATG-AGC 353
                * ***                                            **:****

Probe11         ------------------TCCG-----------TCACC---------------------- 39
CDC42EP5        TTTCTATGACGGAGTGGCTCCAGGCAGGGAGGTTCATCTAGGGGACG-CCGCAGCAAAGC 412
                                  ***.           *** *

Probe11         CTGAGCAACC-------------------------------------------------- 49
CDC42EP5        CAGAGCTGCCCCTCTCAGACACCCTCCATGCTCTTCAT-CCTCCTGCTGCTTTCACTGTC 471
                *:****:.**


Probe11         -------------------------------------------------GCCTTGGCT-- 9
ZFPM1           GTGGGGCTCTTGAA-CACACCCCAGAGGGCAACAGTTCTCCATTTCCAGGCCTGAGCTGA 295
                                                                 **** .***

Probe11         -AAAA----------------------------GACTGATTCAAGT------TATG---- 30
ZFPM1           CAGCA-CCGCGCCCACAAACCTCGCTGACCTCTGACTGCTCCGAGAGGGGGCTGTG-GCC 353
                 *..*                            *****.* *.**:      *.**

Probe11         -----------------TCCGTC------------------ACCCTG------------- 42
ZFPM1           CGGGCGCTGTGGGCAACTCCTTCGTGGGTCTCCTGCGGGGCGCCCTG-GAGAGGCTCGGT 412
                                 *** **                  .*****

Probe11         -------------------AGCAAC-C--------------------------------- 49
ZFPM1           GAGGAAAGAGGCCCCAAGTGGCAACGCCCAGGGCTGCC-GGCTCCCGCGCCCCCTCCTCG 471
                                   .***** *


Probe11         -----------------G---------CCTTG----------GCTA-------------- 10
SOX1            CAACCGGTTGT-GGTGCGGCCACATGTCCCTGGAGAGTGTCAGCAAGACGGGTCGACTTC 648
                                 *         ** **          **:*

Probe11         -------------AAAGACTG------------------ATTCAA------GT------- 26
```

```
SOX1            AC-CAGGACCACGAAAGACAGGGCAGAGTTTCTGTCTTCTTTCACTGTCCCGT-AGAACT 706
                             ******:*                  :****.      **

Probe11         --TA---TGTCCGTC---------------------------ACCCTGAGCAA------C 48
SOX1            CCTAGCTTGTCCATCGTGCTCCTTGCTTGGGACTCGGGACTGACACTGGGCCAGAAAATC 766
                  **   *****.**                           **.***.**.*      *

Probe11         C----------------------------------------------------------- 49
SOX1            CACCTCTAGATGCCTCAAAGCTCGCTTTCATGCTGGGGGGCGGGCGGGAGCGGGGAAGGG 826
                *



Probe11         ---------------------G----CCTTGG-----------CTAAAAGACTG------ 18
CASKIN1         AATCCCTCGACACCTCCCACGGGAACCCTGGG-CCAGGAACGCCTGACAGCCTGGCGGGC 177
                                     *    *** **           **.*.**.***

Probe11         --ATTCAAG---TTATG------------------------------------------- 30
CASKIN1         CGGCTCCAGGGGCACTGGCGCCC-GGGTCTCTCGGCGCCCGGTCCCCGCCCGCCCGCTGC 236
                  . **.**    :.**

Probe11         ------TCCGTCAC---------------------------------------------- 38
CASKIN1         CTCGGAACCGTCCC-CCGCGGGTCCCGCAGCCTCTGGAAGCCTCTCTGGGCTCAGCCCCG 295
                      :*****.*

Probe11         -----------------------CCTGAGCAACC-------------------------- 49
CASKIN1         CGCCG-CCGCCGCGTCGGGGCTCCCAGTGCACCCCCGGCGGCCGCGTCCCCAGCCC-GGC 353
                                       **:*:***.**



Probe11         ----------------------------------------------GCCTTG-------- 6
SOX3            CAACGGGAAGCTTT-GCGGGCTGGCCGGGGGATGGGGCGCCGGTCTGCCTTGACAGGGTT 295
                                                              ******

Probe11         GCTAA-------------------AAG-------ACTG------------ATTCAA-GTT 27
SOX3            GCAAA-GTTGTTTTCTAAATTCCGAAGGCGCCCCTCTGCCCCCTCCCCCCAATCTG-CTT 353
                **:**                   ***       :***            *:**:.  **

Probe11         ATGT------------------CCGTCACC------------------------------ 39
SOX3            GCGTGCCCCCTCCCCCCTCCCCCCGTCACCTCCTCAGGTTTCGTTCT-TTCAAACTTTTT 412
                . **                  ********

Probe11         --------------------CTGAG--------------CAACC---------------- 49
SOX3            GAAACCCTAATTGGTGGCCTCTGAGTGGGCCTCGTGGA-CTCCCGCCTCCTAAGTAACTC 471
                                    *****              *:.**



Probe11         -------------------------------------------GCCT------------- 4
KLF16           GGAGTGGGCGCGCCGGGGGGCGGGTTCCTCCGGCCCGAGCCCCGCCC-CCGGCGGCCGCG 412
                                                           ***

Probe11         --TGGCTAAAAGACTGATT----------------------------------------- 21
KLF16           CGCGGCTCGGAGAGTGGTGGAGGGCGCGCCGGGCGGGG-GGCGTGGCTGCGGCGCCCCGA 471
                   ****   *** ** *
```

AK-Supplementary Figure S3

```
Probe11         ---------------------CAAGTTAT---------------------GTCCGTCA-- 37
KLF16           GGGGGCGTCCTCCGGGCGGGGCGGGCTGT-GGCCGCCCCCGCGGGCTCCGGTGCGTCAGG 530
                                     *  * * *                     ** *****

Probe11         ------------------------------------------------------------
KLF16           ACGCGTCAGGCGGGGCGGGG-CAGGCCGAGCGCGGGCGTCGGCGGCGGCGCGCGCCGGGC 589


Probe11         -------CCCT----------------------GAGCAACC------------------- 49
KLF16           CTCCTCCTCCT-CCTCCTCCGCCTCCCGCACTCGAGCAGCCGCCGCCGGCCGGACGGACG 648
                        ***                      ***** **
```

Supplementary Figure S3. Alignment of the probe 11 sequence with promoters sequences of TSC2
regulated genes by the ClustalW2 program. Only the portions of promoter sequences aligning with the probe 11 sequence are shown.
