# Supplementary material for: Evidence that TSC2 acts as a transcription factor and binds to and represses the promoter of Epiregulin
Source: Nucleic Acids Res. 2014 Apr 19;42(10):6243–55. doi: 10.1093/nar/gku278 (PMC4041451; doi:10.1093/nar/gku278)
Supplement: SUPPLEMENTARY DATA [file supp_gku278_nar-03278-v-2013-File014.pdf]

Supplementary Table S1. PCR primers used for the validation of stable clones and microarray data.

Gene	Accession no.	Primer sequence (5’-3’)	T_m (°C)	Amplicon size (bp)	Reference
RPL35A	NM_000996.2	F: GGGTACAGCATCACTCGGA<br>R: ACGCCCGAGATGAAACAG	58	220	(15)
TSC2	X75621	F: CCGTCTTCCACATCGCCACCCTG<br>R:ACGATCACGTGGACAAAGTTGAACTG	67	168	(25)
ANO2	NM_020373.2	F: AAGTGCGGCTCGATGCAAAGAAG<br>R: CGGAGGTGATCGCAATGACAAAAG	62	152	-
MAPK4	NM_002747	F: TCTTCATCAGCACAGAGGACCTC<br>R: GGAGCAGTCGTGGGGAACGG	62	138	-
EREG	NM_001432	F: ACAACTGTGATTCCATCATGTATCC<br>R: CTACACTTTGTTATTGACACTTGAGC	62	109	-
UCA1	NR_015379.2	F: GAGCTTCGGGTAACTCTTACGG<br>R: GATGGACGGCAGTTGGTGTGC	62	153	-
TSC2 (TAD1)	X75621	F: TGTGCAGGAGAAGACGAACC<br>R: GGCACCGACAGTGACTTGTA	58	240	-
TSC2 (TAD2)	X75621	F: GGTGTCCCTGCAGTGCAGGAAA<br>R: GAGCCGCTTGATGTGGCGGAG	56	205	-

Abbreviations: F, forward primer; R, reverse primer; T_m, annealing temperature; and, bp, base pairs

Supplementary Table S2. Details of PCR primers used in the generation of different deletion constructs.

Construct	Primer sequence (5’ – 3’)	T_m (°C)	Amplicon size (bp)	Reference
pFP1EREG	F:TAGCCTCGAGCTCCATCAGCATAGGCAGGAA<br>*Xho*I<br>R:TAGCAGATCTCGGCGATGGGAGCGGGCGCT<br>*Bgl*II	53	1022	(17)
pFP2EREG	F:TAGCCTCGAGACAATGACTCGACTTTGAGG<br>*Xho*I<br>R:TAGCAGATCTCGGCGATGGGAGCGGGCGCT<br>*Bgl*II	58	733	(17)
pFP3EREG	F:TAGCCTCGAGCACTACTCTCAGGTGCTCCAG<br>*Xho*I<br>R:TAGCAGATCTCGGCGATGGGAGCGGGCGCT<br>*Bgl*II	58	328	(17)
pFP4EREG	F:TAGCAGATCTCTCCATCAGCATAGGCAGGAA<br>*Bgl*II<br>R:TAGCAAGCTTCCTCAAAGTCGAGTCATTGTGA<br>*Hind* III	58	309	-
pFP1EREG-mut	F:ACCACCCACTACAGGGCAGG<br>R:GTTCCCCATACATGCCTTTGGA	56	966	-
pcDNA3.1(+)/TSC2-NLSdel	F:GAGGAAGCCGCCTACTCCAAC<br>R:GGCAATCCACTTGGAGGGGTAG	58	5385	-

Supplementary Table S3. PCR primers used for the chromatin immunoprecipitation (ChIP) analysis.

Primer	Pimer sequence (5' – 3')	T_m (˚C)	Amplicon size (bp)	Reference
EREGF	CTCCATCAGCATAGGCAGGAA	57	554	-
EREGR	GGTTGCTCAGGGTGACGGACATA			
EREGF1	TATGTCCGTCACCCTGAGCAACC	57	491	-
EREGR1	CGGCGATGGGAGCGGGCGCT			
SP1F	GGTACCGCTCCAAGGTTGCCTTCTCTATGA	56	601	(19)
SP1R	CTCGAGGGCCAACCTCGGCCCTTCCTG			

Supplementary Table S4. Sequences of the probes used in EMSA.

Oligo	Sequence(5' - 3')	Size (bp)	Probe*
TS1F<br>TS1R	CTCCATCAGCATAGGCAGGAAAGTGGGAGTGAGGAAAGGCTTAGAGTTTT<br>AAAACTCTAAGCCTTTCCTCACTCCCACTTTCCTGCCTATGCTGATGGAG	50<br>50	Probe 1 (-857 bp to -807 bp)
TS2F<br>TS2R	CCAGCCTGGCTGGGGTCAGACAATTTGCCTGGGGAAATAGTGGGAGCTGA<br>TCAGCTCCCACTATTTCCCCAGGCAAATTGTCTGACCCCAGCCAGGCTGG	50<br>50	Probe 2 (-807 bp to -757 bp)
TS3F<br>TS3R	GGTTGAAAAGACCGATTGGCTTCACGTTTGTGGCAGGCCTTGTCCAAAAAG<br>CTTTTTGGACAAGGCCTGCCACAAACGTGAAGCCAATCGGTCTTTTCAACC	51<br>51	Probe 3 (-757bp to -706 bp)
TS4F<br>TS4R	CAATAGGAAGTCGTTGAAAGGTTTTGTGCAGGGGAGCGACAGGATTAAGG<br>CCTTAATCCTGTCGCTCCCCTGCACAAAACCTTTCAACGACTTCCTATTG	50<br>50	Probe 4 (-706 bp to -656 bp)
TS5F<br>TS5R	CACTACTTAGTAGGAAGTCATATCAAAAGCAGCAACCTGACAAATGGCAAA<br>TTTGCCATTTGTCAGGTTGCTGCTTTTGATATGACTTCCTACTAAGTAGTG	51<br>51	Probe 5 (-656 bp to -605 bp)
TS6F<br>TS6R	TACGACAGAAGGAAAAAAAATAACAGGAATTTTCTTCACAATGACTCGACTT<br>AAGTCGAGTCATTGTGAAGAAAATTCCTGTTATTTTTTTTCCTTCTGTCGTA	52<br>52	Probe 6 (-605 bp to -553 bp)
TS7F<br>TS7R	TGAGGTAATAAATGCCTGAGCCACATTGGAAGCAGGGGTCTCAGAAGGAA<br>TTCCTTCTGAGACCCCTGCTTCCAATGTGGCTCAGGCATTTATTACCTCA	50<br>50	Probe 7 (-553 bp to -503 bp)
TS8F<br>TS8R	GGCGCAGATGGGAAAGGCTTTTCTGGGCGGTGGCGTTTCAATGCAAGGAG<br>CTCCTTGCATTGAAACGCCACCGCCCAGAAAAGCCTTTCCCATCTGCGCC	50<br>50	Probe 8 (-503 bp to -453 bp)
TS9F<br>TS9R	GGCGCTTGAATCACTGACAGACTTCAAGTTAAGGGAGTTTTCGTGGCTGAG<br>CTCAGCCACGAAAACTCCCTTAACTTGAAGTCTGTCAGTGATTCAAGCGCC	51<br>51	Probe 9 (-453 bp to -402 bp)
TS10F<br>TS10R	GTTAGTAAGTCACACGCACAGCTCTCCAAAGGCATGTATGGGGAACGCCA<br>TGGCGTTCCCCATACATGCCTTTGGAGAGCTGTGCGTGTGACTTACTAAC	50<br>50	Probe 10 (-402 bp to -352 bp)
TS11F<br>TS11R	GCCTTGGCTAAAAGACTGATTCAAGTTATGTCCGTCACCCTGAGCAACC<br>GGTTGCTCAGGGTGACGGACATAACTTAATCAGTCTTTTAGCCAAGGC	49<br>49	Probe 11 (-352 bp to -303 bp)

***** A double-stranded probe was made by mixing equal amount of each of the two oligos.
Abbreviation: bp, base pairs.
